# Presence of a widely disseminated *Listeria monocytogenes* serotype 4b clone in India

**DOI:** 10.1038/emi.2016.55

**Published:** 2016-06-08

**Authors:** Sukhadeo B Barbuddhe, Swapnil P Doijad, Alexander Goesmann, Rolf Hilker, Krupali V Poharkar, Deepak B Rawool, Nitin V Kurkure, Dewanand R Kalorey, Satyaveer S Malik, Ingudam Shakuntala, Sandeep Chaudhari, Vikas Waskar, Dilecta D'Costa, Rahul Kolhe, Ritu Arora, Ashish Roy, Abhay Raorane, Satyajit Kale, Ajay Pathak, Mamta Negi, Simranpreet Kaur, Rupesh Waghmare, Shubhangi Warke, Shabu Shoukat, Belgode Harish, Aruna Poojary, Chakodabail Madhavaprasad, Karabasanavar Nagappa, Samir Das, Ravindra Zende, Sandeep Garg, Saroj Bhosle, Savio Radriguez, Ashish Paturkar, Moritz Fritzenwanker, Hiren Ghosh, Torsten Hain, Trinad Chakraborty

**Affiliations:** 1 National Institute of Biotic Stress Management, Raipur 493 225, India; 2 Institute for Medical Microbiology, Justus-Liebig University of Giessen, 35392 Hesse, Germany; 3 Systems Biology, Justus-Liebig University of Giessen, 35392 Hesse, Germany; 4 Nagpur Veterinary College, Nagpur 440006, India; 5 Division of Veterinary Public Health, Indian Veterinary Research Institute, Izatnagar 243 122, India; 6 Indian Listeria Consortium, Center of Excellence and Innovation in Biotechnology on Molecular Epidemiology of Listeria Monocytogenes, National Institute of Biotic Stress Management, Raipur 493 225, India

**Dear Editor,**

*Listeria monocytogenes* is an important foodborne pathogen with a high fatality rate. Clinically, infections are associated with particular high-risk groups, including unborn, newborn, elderly and immuno-compromised individuals. The majority of listeriosis cases are caused by serotypes 1/2a, 1/2b and 4b, and outbreaks and cases attributable specifically to serotype 1/2a are increasing in both the United States and Europe. In India, infections with *L. monocytogenes* remain largely undiagnosed and are under-reported both because of a lack of awareness and the limited availability of proper diagnostic assays. In an Indo-German collaboration, we collected 830 listerial strains comprising different listerial species isolated during 2000–2014 in the Indian Listeria culture collection (ILCC). Further analysis identified 396/830 strains as *L. monocytogenes* represented by serotypes 4b (*n*=239), 1/2a (*n*=110) and 1/2b (*n*=47; Supplementary Table S1).^1, 2^ The data indicated an overall preponderance of serotype 4b strains from different sources and geographically dispersed regions.

On the basis of their availability, two representative strains of each serotype (that is, 4b, 1/2a and 1/2b) from each source (that is, animals, humans, foods and the environment) of each geographical region (*n*=21) were selected to cover the spatial strain diversity. A total of 98 strains (4b *n*=53, 1/2a *n*=23 and 1/2b *n*=22) were analyzed with pulsed-field gel electrophoresis (PFGE; Figure 1A). The PFGE analysis revealed diverse patterns, particularly for the strains belonging to serotypes 1/2a and 1/2b. In contrast, 37 out of the 53 serotype 4b strains obtained from different sources and geographical locations over a period of 14 years exhibited a single indistinguishable PFGE pattern (designated herein as Ind-4b-dom-pulsotype; Figure 1A). To further establish whether the majority of the serotype 4b strains from India were clonal, an additional 56 serotype 4b strains from different sources and geographical regions were analyzed. Of these, 38/56 strains exhibited PFGE patterns indistinguishable from those of the ‘Ind-4b-dom-pulsotype' (Supplementary Figure S1A). Thus, altogether, 68.80% serotype 4b strains exhibited identical pulsotypes.

To further determine the molecular basis of the relatedness of these strains, we performed whole-genome sequencing of 11 strains from human-clinical (*n*=6), animal-clinical (*n*=3), food (*n*=1) and atypical (mosquito; *n*=1) sources (Supplementary Table S2). These strains were well discriminated temporally (isolated between 2001 and 2014) and spatially (from eight different regions). Six of the 11 sequenced strains were also analyzed for their virulence potential by using *Galleria mellonella* larvae and were deemed to be pathogenic (Supplementary Figure S2).

The characteristics of the genomes of the sequenced strains are summarized in Supplementary Table S2. On average, a sequencing coverage of 98.85% (98.21%–99.26%) was obtained (using *L. monocytogenes* F2365 as the reference strain). Comparison of the 11 genomes revealed that 2651/2717 genes (excluding those of the prophages) were common. When directly compared with the F2365 strain, between 29 and 39 genes per strain were either absent or truncated. Strain-specific genes were rare at between zero and four genes per isolate (Supplementary Table S3). Most of the deletions/truncations and additions of genes were due to point mutations.

In combination with 27 previously published *L. monocytogenes* genomes, the phylogenetic context of the sequenced strains was determined on the basis of the amino acid sequences of a concatenated set of 2401 core genes (Supplementary Figure S3). We also included the genome sequences of three serotype 4b strains from India, which were available from an independent study, for comparison.^3^ All of the sequenced strains in this study and the strains from the independent study grouped together with *L. monocytogenes* F2365, which is a well-characterized outbreak strain from the Jalisco soft cheese outbreak in 1986/7 in the United States, and shared 2747 genes with this strain. A total of 18 genes, including the gene for the internalin B protein, were truncated in the outbreak-causing F2365 strain;^4^ however, all of these genes were intact in the strains sequenced in this study. In addition, the average nucleotide identity of all of these serotype 4b strains, including three strains from an independent study, was 99.99%, thus supporting their highly clonal nature.

To examine the similarity at the nucleotide level, we performed comparative single nucleotide polymorphism (SNP) analyses of the sequenced strains against the F2365 reference strain. A total of 377 SNPs were observed, and 83 of these SNPs were commonly present (Supplementary Table S4). The individual strains exhibited between 155 and 230 unique SNPs per strain. Compared with F2365 and with the exception of the prophage insertions, the few discrepancies based on the synteny of the genomes of the clonal strains were apparently due to the occurrence of these putative transposases. These transposases were similar in sequence and were present as inverted elements in the various genomes examined in this study (Supplementary Table S4, Supplementary Figures S4 and S5). The regions flanking these elements were variable and identified as hotspots for SNPs and deletions, which indicated microevolution amongst the strains. Overall, the conservation of the genome was remarkable, given that the strains were obtained from over an extended period (13 years) from different isolation sources and diverse geographical locations.

Prophages were observed in the seven of the 11 strains. Two prophages of 37.8 and 37.6 kb were inserted at identical locations in five strains, that is, ILCC004, ILCC026, ILCC028, ILCC271 and ILCC607. ILCC616 harbored only the 37.8 kb bacteriophage. These bacteriophages are similar to the previously described *Listeria* phages LP-030-02 and LP-030-03 and are members of the family *Myoviridae.*^5^ All of the detected prophages were identical in terms of nucleotide sequences and sizes. The only exception was observed in ILCC619, in which three different prophages with sizes of 16.25, 36.5 and 36.96 kb were detected at discrete locations. Thus, some of these clonal strains could be differentiated only on the basis of the types and locations of the prophages.

To permit comparisons with known *L. monocytogenes* strains, we performed backward compatibility studies by determining both the multilocus sequence types (MLSTs) and multi-virulence-locus sequence types (MVLSTs) of all the strains. The MLST analysis revealed that 10 of the 11 strains were sequence type (ST) 328, and one strain was ST1. ST328 is a single locus variant of ST1 that was assigned to a strain from 1936 and is representative of clonal complex I (CC1) of *L. monocytogenes* (Supplementary Figure S6). Interestingly, we noted that of the nine previously reported ST328 strains in the Listeria-MLST database that were from an independent study, eight were from India and a single strain was from Australia.^6^ These eight ST238 strains were isolated from the environment and foods. Among the seven CCs that had previously been demonstrated to be associated with clinical cases, CC1 was slightly more diverse, with a central genotype that exhibited two single locus variants with additional variants (Supplementary Figure S6).^7^ According to surveys of isolates obtained from foods, food processing plants and other habitats, CC1 strains are apparently ubiquitous in the environment and have repeatedly been introduced into foods,^8^ thus probably reflecting the greater fitness of the CC1 strains in the environment^8^ and possibly accounting for the frequent isolation of CC1 strains throughout the country.

The MVLST analyses indicated that these strains were of the virulence profile type 20 (VT20) and were members of the epidemic clone I group (Supplementary Figure S7). In a previous study, we have found that 84% of all serotype 4b strains from ILCC belonged to VT20, thus supporting the persistence of a single clone.^9^ Strains of the CC1/VT20 group have previously been implicated in foodborne outbreaks in Nova Scotia (1981), Switzerland (1983–1987), the United States (1986 and 1987) and France (1992).^10^ The PFGE profiles (*Apa*I and *Asc*I) of the strains for a given CC can vary considerably.^10^ In contrast, however, the PFGE profiles of the CC1 strains in this study were highly homogeneous, thus suggesting robustness and supporting their clonal nature. Moreover, the three serotype 4b strains from the independent study^3^ have also been observed to be of the MLST ST328 (CC1) and MVLST VT20 varieties, thus supporting the spread or persistence of the clonal serotype 4b strain in India.

In conclusion, our study provides strong evidence for the circulation of a stable and widespread epidemic clone of *L. monocytogenes* serotype 4b in the Indian subcontinent (Figure 1B). The routes of transmission of this clone (‘Ind-4b-dom-pulsotype', ST328, VT20), which is geographically unique and distributed over enormous distances in both space and time, are presently unknown. The frequency of the isolation of this pathogenic major clone from different sources warrants further attention and indicates the need for further studies of its influence on public health and foodborne diseases in India. The occurrence of ST328 appears to be restricted primarily to India, and investigations into its presence in different countries, particularly those in East and Southeast Asia, should be conducted.

Materials and methods are provided in Supplementary Data.

## Figures and Tables

**Figure 1:**
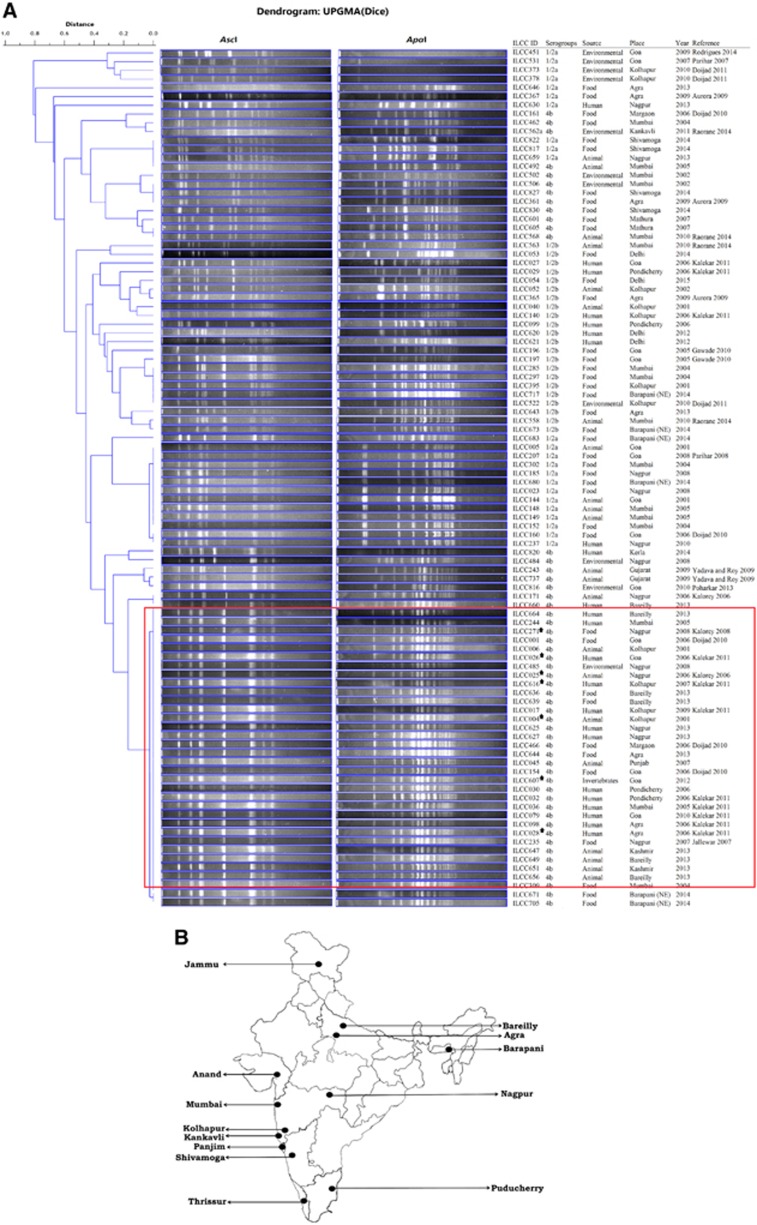
(**A**) *Asc*I and *Apa*I enzyme-generated pulsed-field gel electrophoresis patterns of 98 representative *L. monocytogenes* strains from different sources, times and geographical regions of India. Among the 4b serotype strains observed in the culture collections, 68.80% exhibited unique pulsotypes and were designated as the Ind-4b-dom-pulsotype (marked with the red square), thus suggesting the persistence of a dominant clone across India. *sequenced strain. (**B**) Geographic locations in India from which the *L. monocytogenes* 4b strains with dominant pulsotypes were obtained. These locations represent the sub-collection centers that independently studied the occurrences of *L. monocytogenes* from different sources within the associated geographical regions.
