# Supplementary material for: Presence of a widely disseminated Listeria monocytogenes serotype 4b clone in India
Source: Emerg Microbes Infect. 2016 Jun 8;5(6):e55–. doi: 10.1038/emi.2016.55 (PMC4932648; doi:10.1038/emi.2016.55)
Supplement: Supplementary Data [file emi201655x1.docx]

**Materials and Methods:**

DNA sequencing libraries were prepared using the Nextera XT kit (Illumina, San Diego) and sequenced on an Illumina MiSeq. The sequences were assembled after quality control with SPAdes, and the resulting contigs were ordered with r2cat,^1^ using the *L. monocytogenes* F2365 serotype 4b as the a standard reference strain and annotated with by GenDB^2^. The SNPs were identified with the determined by BWA tool and ReadXplorer with the thresholds of a the minimum base quality of 20 and a 90% SNP-frequency with a minimum 10 fold coverage for a particular base.^3^ The core-genes, pan-genes and core genome phylogeny were calculated with by ‘Efficient Database framework for comparative Genome Analyses using BLAST score Ratios’ (EDGAR).^4^ The genomes were also analyzed for the analysed for multi-locus sequence types (MLSTs)^5^, multi-virulence locus sequence types (MVLSTs)^6^ and average nucleotide identities (ANIs)^7^.

**References:**

1 Husemann P, Stoye J. r2cat: Synteny plots and comparative assembly. *Bioinformatics* 2009; **26**: 570–571.

2 Meyer F, Goesmann A, McHardy AC, Bartels D, Bekel T, Clausen J *et al.* GenDB - An open source genome annotation system for prokaryote genomes. *Nucleic Acids Res* 2003; **31**: 2187–2195.

3 Hilker R, Stadermann KB, Doppmeier D, Kalinowski J, Stoye J, Straube J *et al.* ReadXplorer - Visualization and Analysis of Mapped Sequences. *Bioinformatics* 2014; **30**: btu205.

4 Blom J, Albaum SP, Doppmeier D, Pühler A, Vorhölter F-J, Zakrzewski M *et al.* EDGAR: a software framework for the comparative analysis of prokaryotic genomes. *BMC Bioinformatics* 2009; **10**: 154.

5 Brisse S. http://www.pasteur.fr/recherche/genopole/PF8/mlst/Lmono.html. http://www.pasteur.fr/recherche/genopole/PF8/mlst/Lmono.html (accessed 20 May2004).

6 Zhang W, Jayarao BM, Knabel SJ. Multi-Virulence-Locus Sequence Typing of Listeria monocytogenes. *Appl Environ Microbiol* 2004; **70**: 913–920.

7 Richter M, Rosselló-Móra R. Shifting the genomic gold standard for the prokaryotic species definition. *Proc Natl Acad Sci U S A* 2009; **106**: 19126–31.

**Details of Members of the Indian *Listeria* Consortium are:

- Ingudam Shakuntala, Samir Das: ICAR Research Complex for NEH Region, Umian, Meghalaya, 793103 India
- Sandeep Chaudhari, Rahul Kolhe, Rupesh Waghmare, Shubhangi Warke, Shabu Shoukat, Ravindra Zende, Vikas Waskar and Ashish Paturkar: Maharashtra Animal and Fisheries Sciences University, Nagpur, 440001 India
- Dilecta D’Costa: Department of Microbiology, Government collge of Khandola, Khandola, 403107 India
- Ritu Arora: Dayalbagh Educational Institute, Agra, 282005 India
- Ashish Roy: College of Veterinary Sciences and Animal Husbandry, Anand, 388 001 India
- Abhay Raorane, Satyajit Kale, Ajay Pathak: ICAR research Complex for Goa, Goa, 403402 India
- Mamta Negi and Simranpreet Kaur: Division of Veterinary Public Health, Indian Veterinary Research Institute, Izatnagar, 243122 India
- Belgode Harish: Jawaharlal Institute of Postgraduate Medical Education and Research, Puducherry, 605006 India
- Aruna Poojary: Breach Candy Hospital Trust, Mumbai, 400026 India
- Chakoda Madhavaprasad and Karabasanavar Nagappa: Veterinary College, Shivamogga 577204 India
- Sandeep Garg and Saroj Bhosle: Department of Microbiology, Goa University, Taleigao Plateau, 403206 India
- Savio Radriguez: Goa Medical College, Bambolim, Goa, 403202 India
