# Supplementary material for: Presence of a widely disseminated Listeria monocytogenes serotype 4b clone in India
Source: Emerg Microbes Infect. 2016 Jun 8;5(6):e55–. doi: 10.1038/emi.2016.55 (PMC4932648; doi:10.1038/emi.2016.55)
Supplement: Supplementary Figure 1 [file emi201655x2.pdf]

Suppl. Fig. 1: *Asc* I PFGE pattern of *L. monocytogenes* serotype 4b strain (set-A, 28 strains)

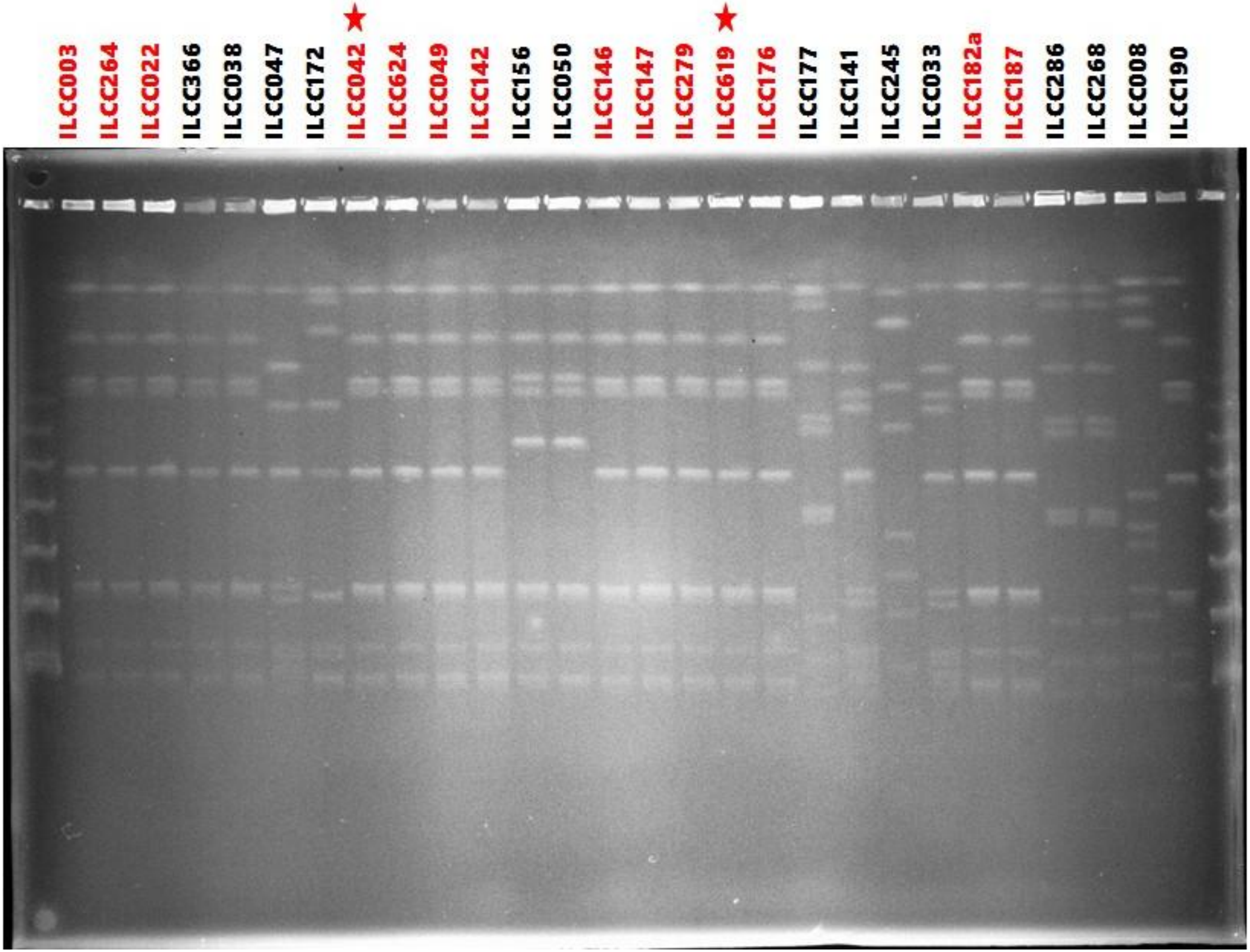

Suppl. Fig. 1 continued: *Apa* I I PFGE pattern of *L. monocytogenes* serotype 4b strain (set-A, 28 strains)

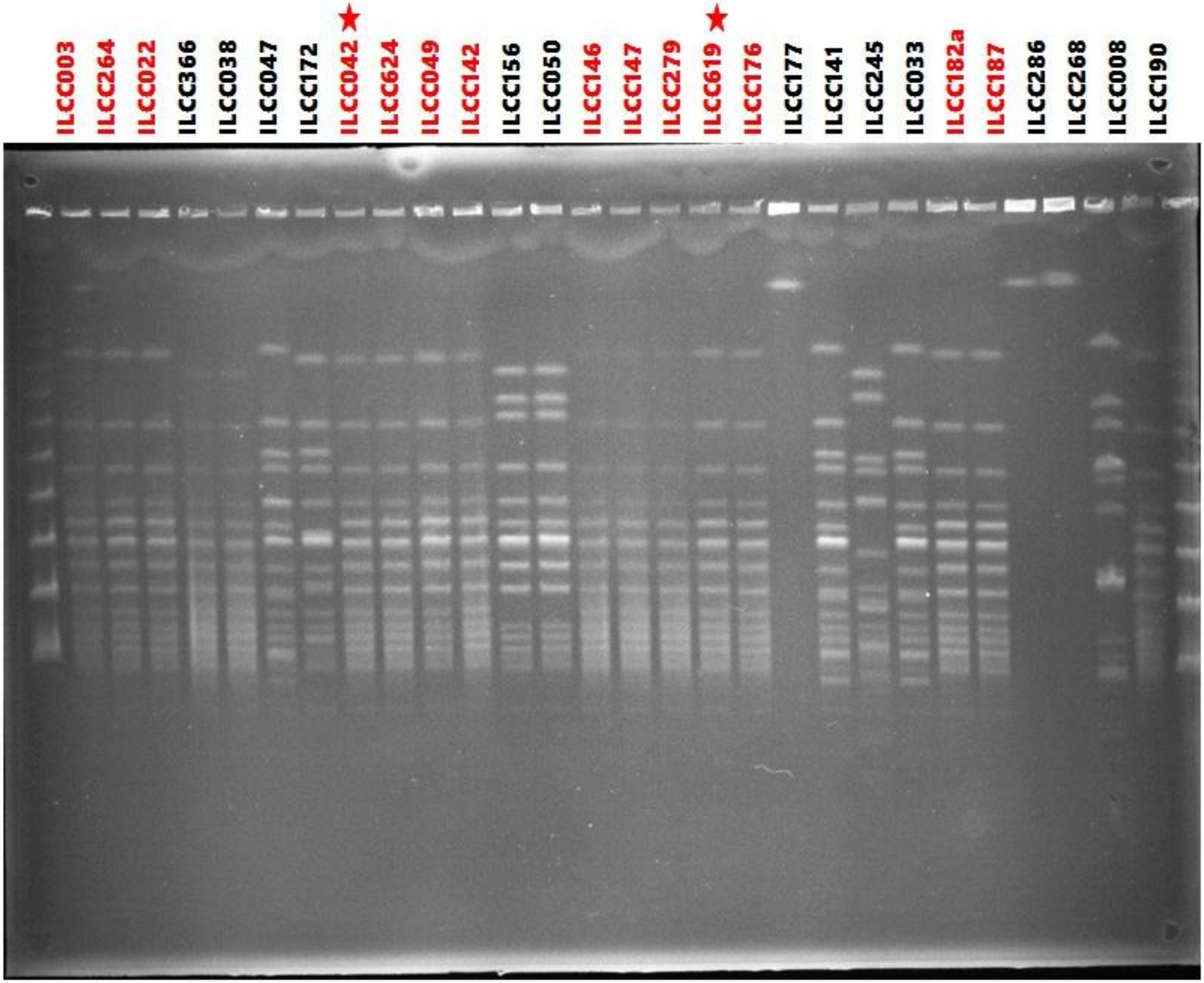

Suppl. Fig. 1 continued: *Asc* I PFGE pattern of *L. monocytogenes* serotype 4b strain (set-B, 28 strains)

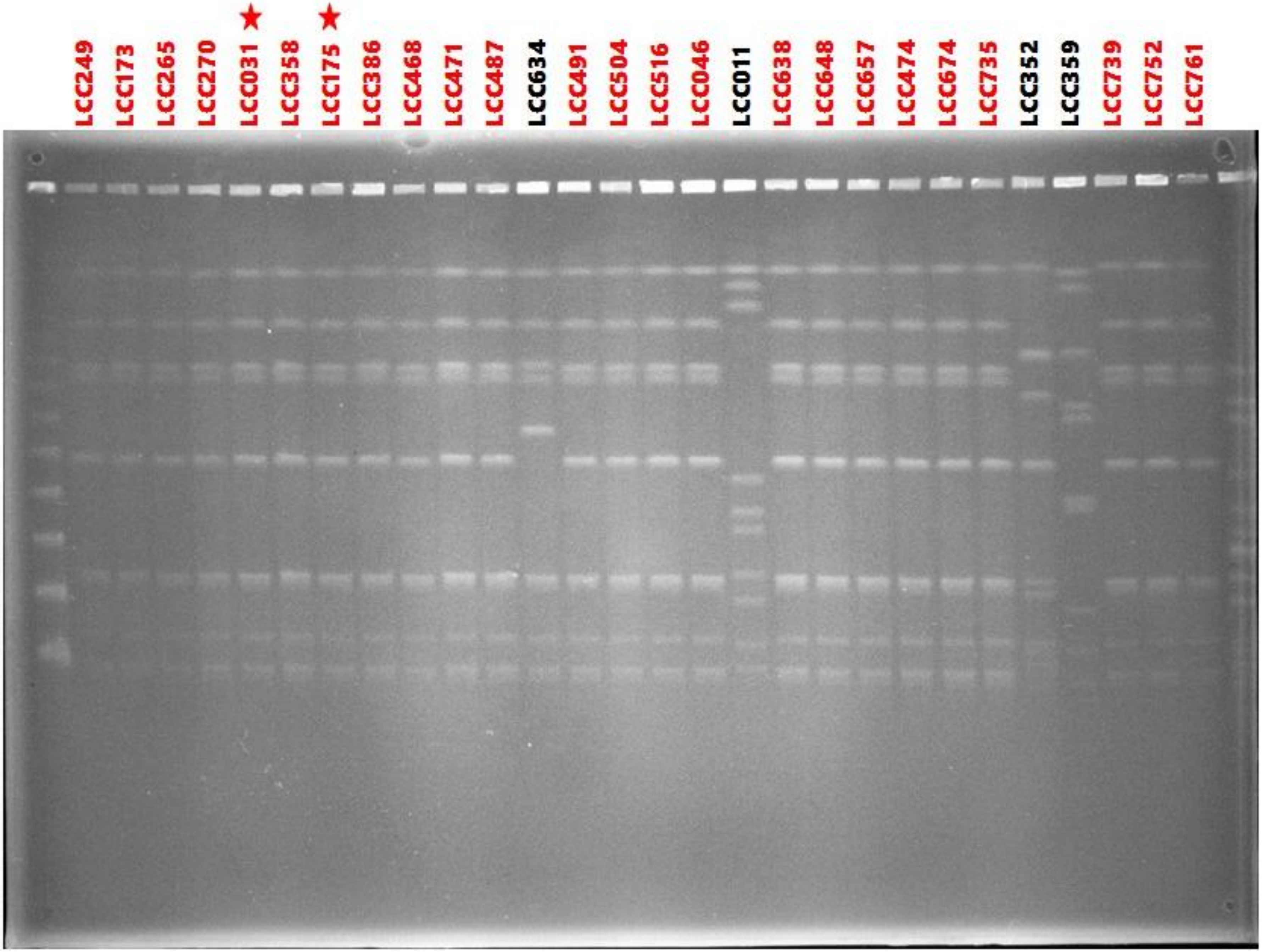

Suppl. Fig. 1 continued: Suppl. Fig. 1 continued: *Apa* I PFGE pattern of *L. monocytogenes* serotype 4b strain (set-B, 28 strains)

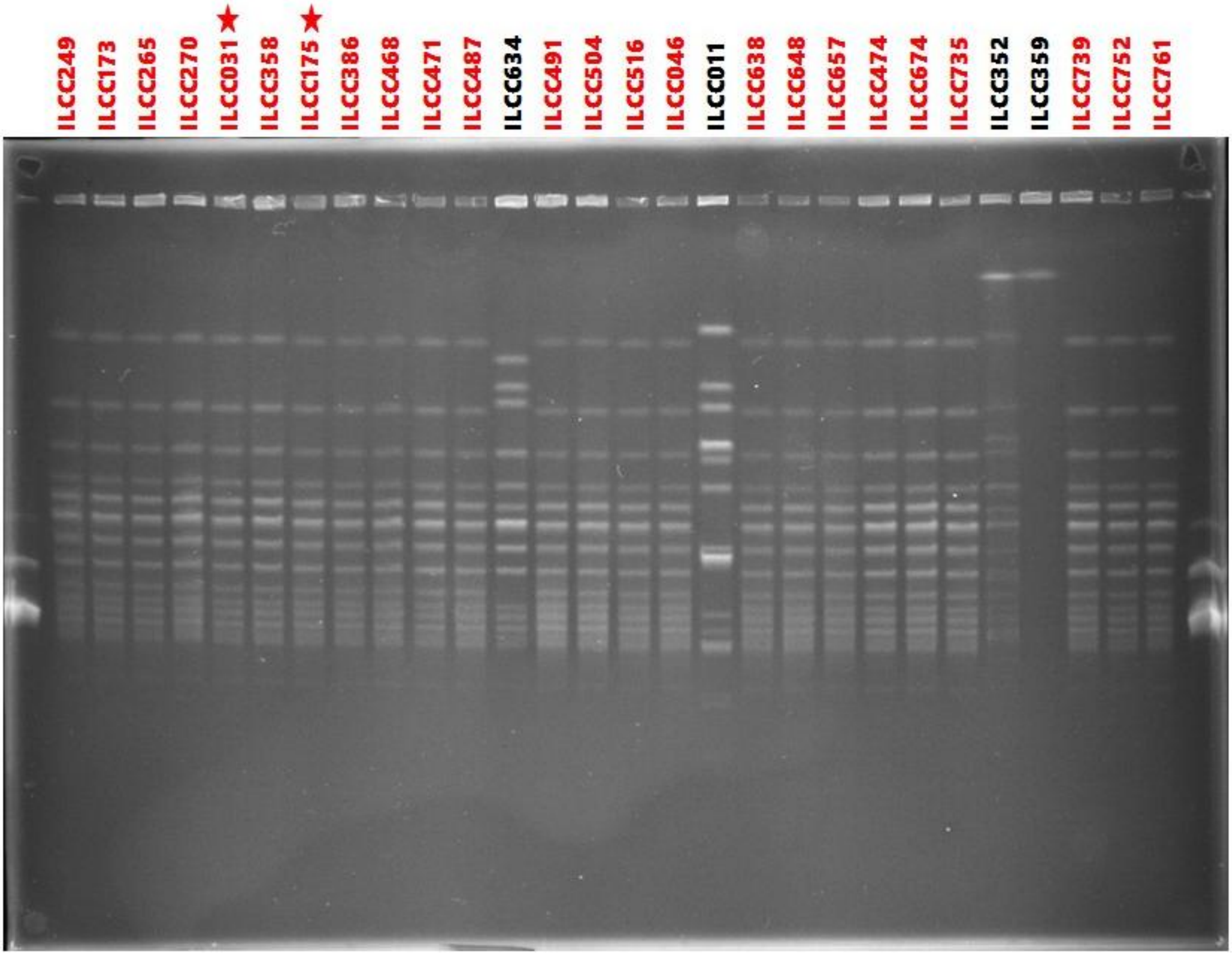

Supplementary Figure S1: *Asc*I and *Apa*I PFGE patterns of the *L. monocytogenes* serotype 4b strains. To confirm that the majority of the *L. monocytogenes* serotype 4b strains present across India are clonal, 56 additional serotype 4b strains were subjected to PFGE, and 39 of these strains exhibited the Ind-4b-dom-pulsotype (marked in red).
