# Supplementary material for: Presence of a widely disseminated Listeria monocytogenes serotype 4b clone in India
Source: Emerg Microbes Infect. 2016 Jun 8;5(6):e55–. doi: 10.1038/emi.2016.55 (PMC4932648; doi:10.1038/emi.2016.55)
Supplement: Supplementary Figure 2 [file emi201655x3.pdf]

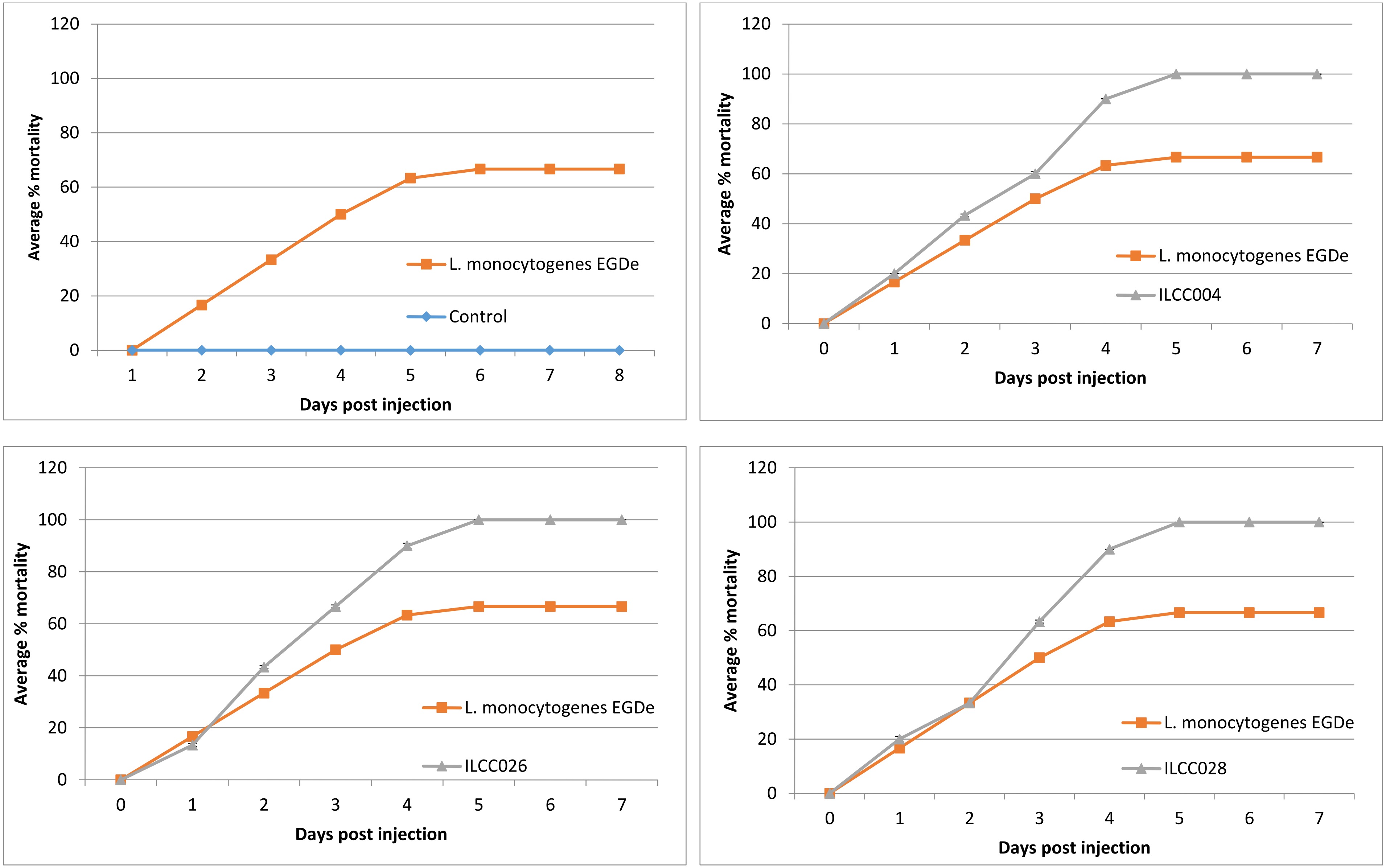

Average % mortality
Days post injection
L. monocytogenes EGDe
Control
ILCC004
ILCC026
ILCC028
120
100
80
60
40
20
0
0
1
2
3
4
5
6
7
8

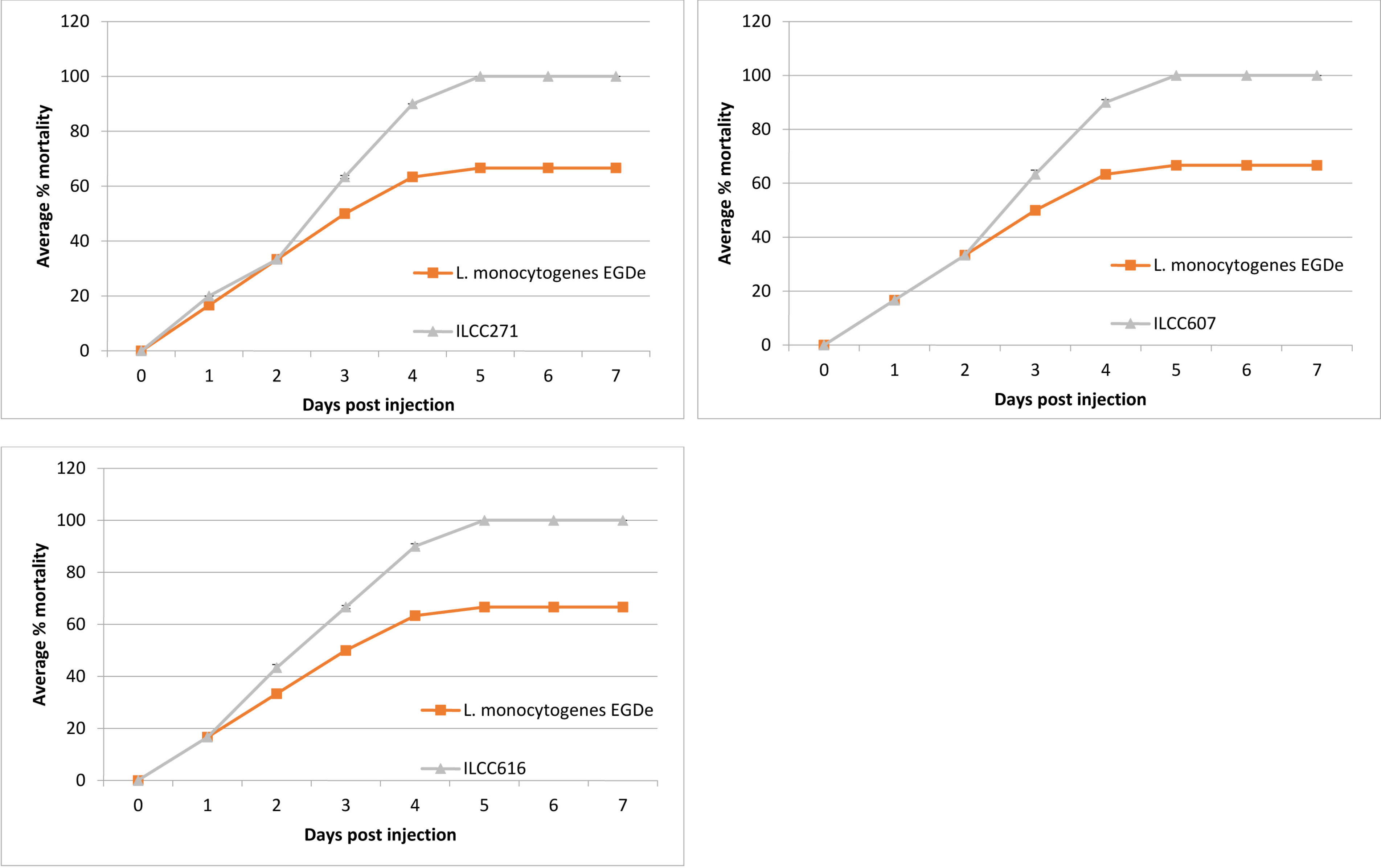


**Supplementary Figure S2** Galleria mellonella mortality chart of six of the clonal L. monocytogenes Indian strains compared with the standard L. monocytogenes EGD-e strain. The six representative L. monocytogenes strains with similar pulsotypes were studied as described by Mukherjee et al. (2010). In brief, ten Galleria larvae per strain were infected with 106 CFU/larvae and observed for mortality for up to 7 days. The experiment was repeated three times, and the average % mortality was calculated. All six of the studied clonal L. monocytogenes 4b sero-type strains were similar in terms of their mortality rates, which were ~20% greater than that of L. monocytogenes EGD-e.
