# Supplementary material for: Presence of a widely disseminated Listeria monocytogenes serotype 4b clone in India
Source: Emerg Microbes Infect. 2016 Jun 8;5(6):e55–. doi: 10.1038/emi.2016.55 (PMC4932648; doi:10.1038/emi.2016.55)
Supplement: Supplementary Figure 3 [file emi201655x4.pdf]

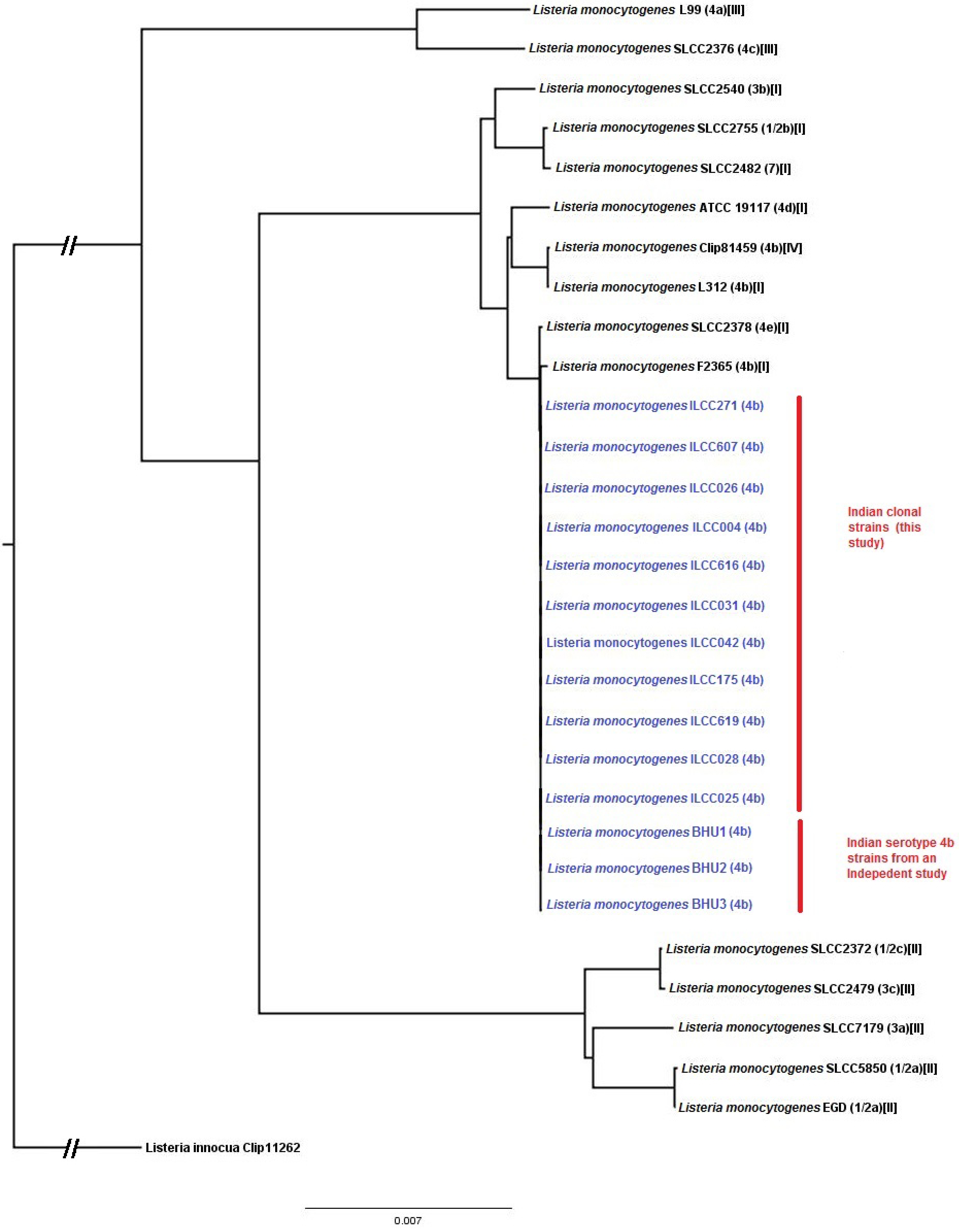


**Supplementary Figure S3.** Neighbor-joining tree based on the alignments of 2401 mutually conserved core genes (amino acid identity >60%, coverage >80%) of L. monocytogenes, including representative strains from every serotype and lineage. The 11 studied representative L. monocytogenes serotype 4b strains from India were observed to be closely related to the outbreak-associated L. monocytogenes F2365 strain that was involved in a cheese outbreak in 1986/7 in the USA. Three L. monocytogenes serotype 4b strains (labeled as BHU01-03) from the independent study from India were included for comparison purposes and found to be identical to the 11 studied strains and F2365.
