# Supplementary material for: Presence of a widely disseminated Listeria monocytogenes serotype 4b clone in India
Source: Emerg Microbes Infect. 2016 Jun 8;5(6):e55–. doi: 10.1038/emi.2016.55 (PMC4932648; doi:10.1038/emi.2016.55)
Supplement: Supplementary Figure 4 [file emi201655x5.pdf]

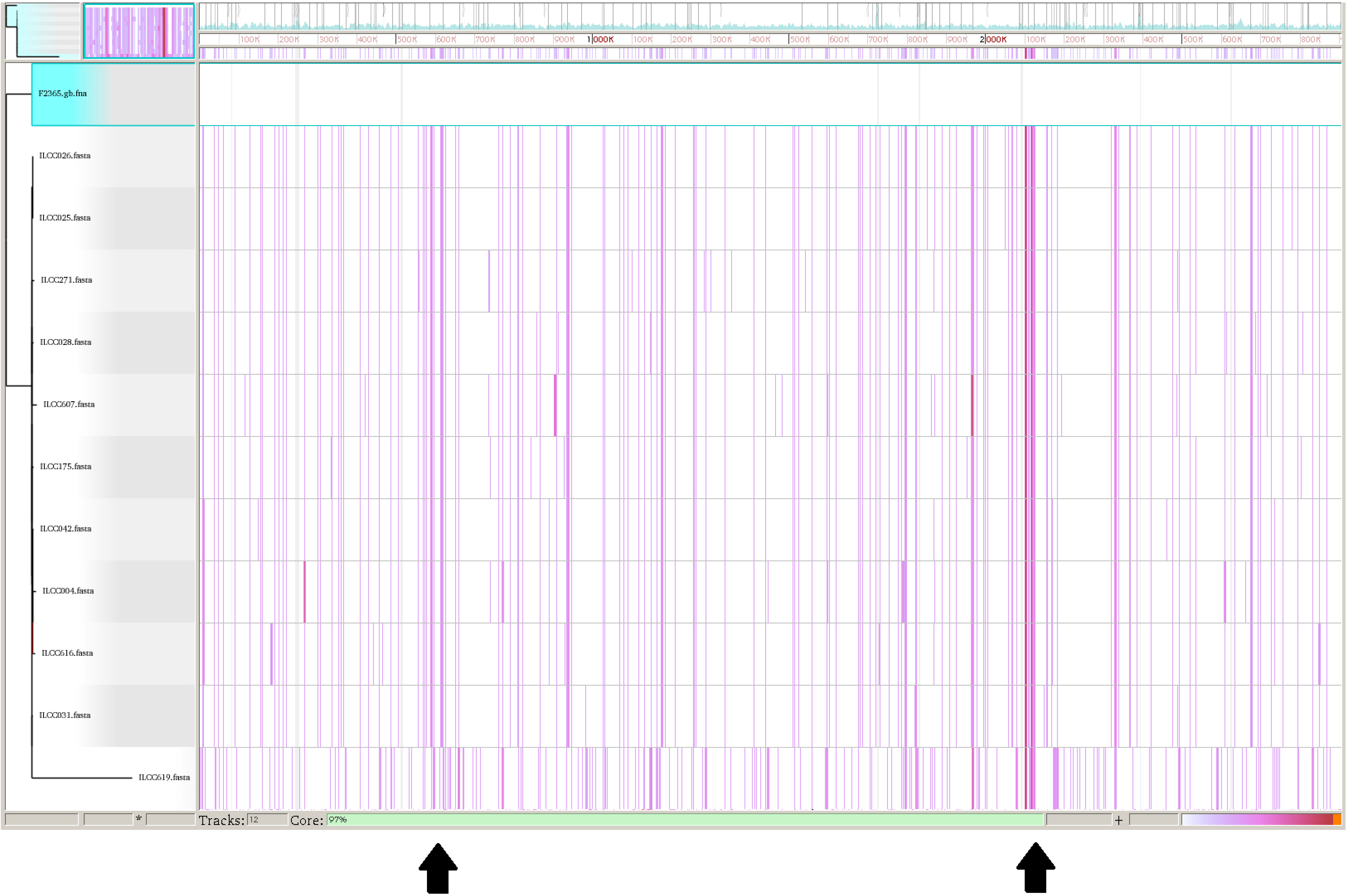

Supplementary Figure 4. Portrait of the SNPs across the genomes compared with the standard reference strain L. monocytogenes F2365. The mapped SNPs were visualized with the Gingr tool(Ref.1). Compared with F2365, a total of 377 SNPs were observed among the Indian strains. Although an average of 1 SNP per ~8 kb was observed across the genomes, >10 per kb of SNPs were observed near the transposases (marked by the arrow).

1. Treangen TJ, Ondov BD, Koren S, Phillippy AM. The Harvest suite for rapid core-genome alignment and visualization of thousands of intraspecific microbial genomes. Genome Biol 2014; 15: 524.
