# Supplementary material for: Presence of a widely disseminated Listeria monocytogenes serotype 4b clone in India
Source: Emerg Microbes Infect. 2016 Jun 8;5(6):e55–. doi: 10.1038/emi.2016.55 (PMC4932648; doi:10.1038/emi.2016.55)
Supplement: Supplementary Figure 5 [file emi201655x6.pdf]

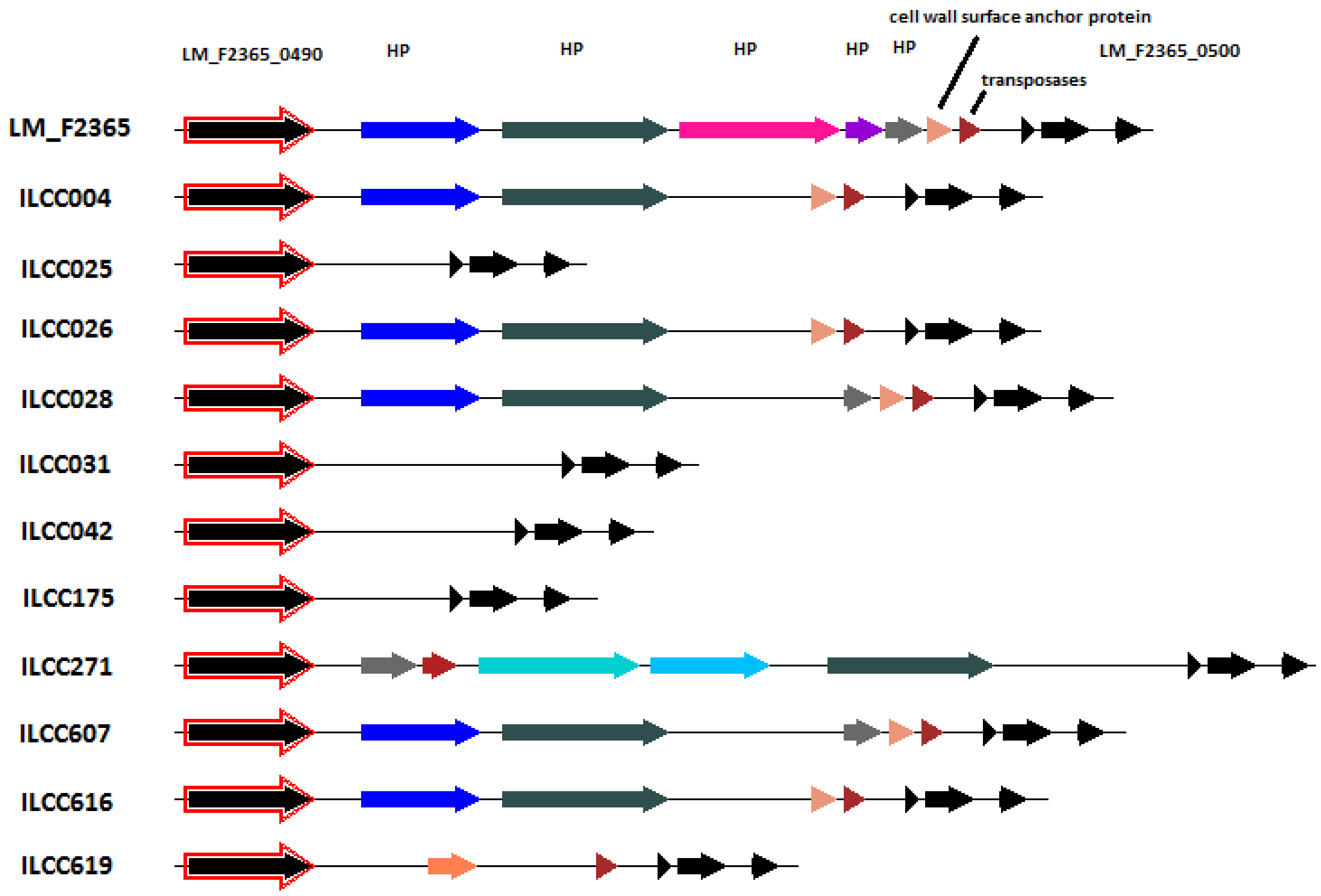

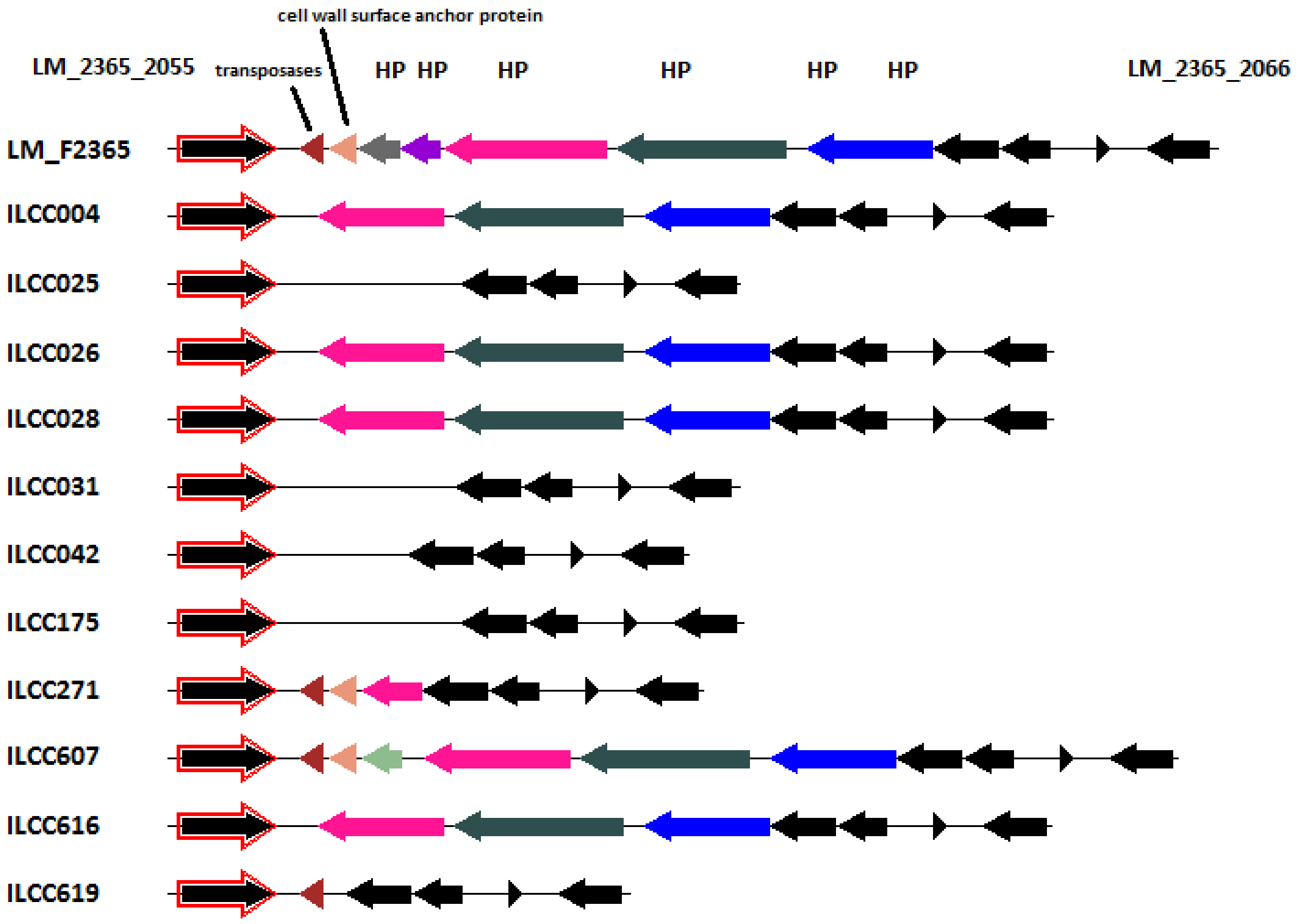

Supplementary Figure 5. Depiction of the genetic rearrangements (gene losses and inversions) among the Indian strains compared with the closely related L. monocytogenes 4b serotype strain. All of the genomes were analyzed and comparatively visualized with the Genome-Comparisons (GECO) comparative genomic tool. (Ref.1) The regions are pegged at positions F2365_0490 and F2365_2055 of L. monocytogenes F2365. HP: hypothetical protein.

1. Kuenne CT, Ghai R, Chakraborty T, Hain T. GECO--linear visualization for comparative genomics. Bioinformatics 2007; 23: 125–126.
