# Supplementary material for: Presence of a widely disseminated Listeria monocytogenes serotype 4b clone in India
Source: Emerg Microbes Infect. 2016 Jun 8;5(6):e55–. doi: 10.1038/emi.2016.55 (PMC4932648; doi:10.1038/emi.2016.55)
Supplement: Supplementary Figure 6 [file emi201655x7.pdf]

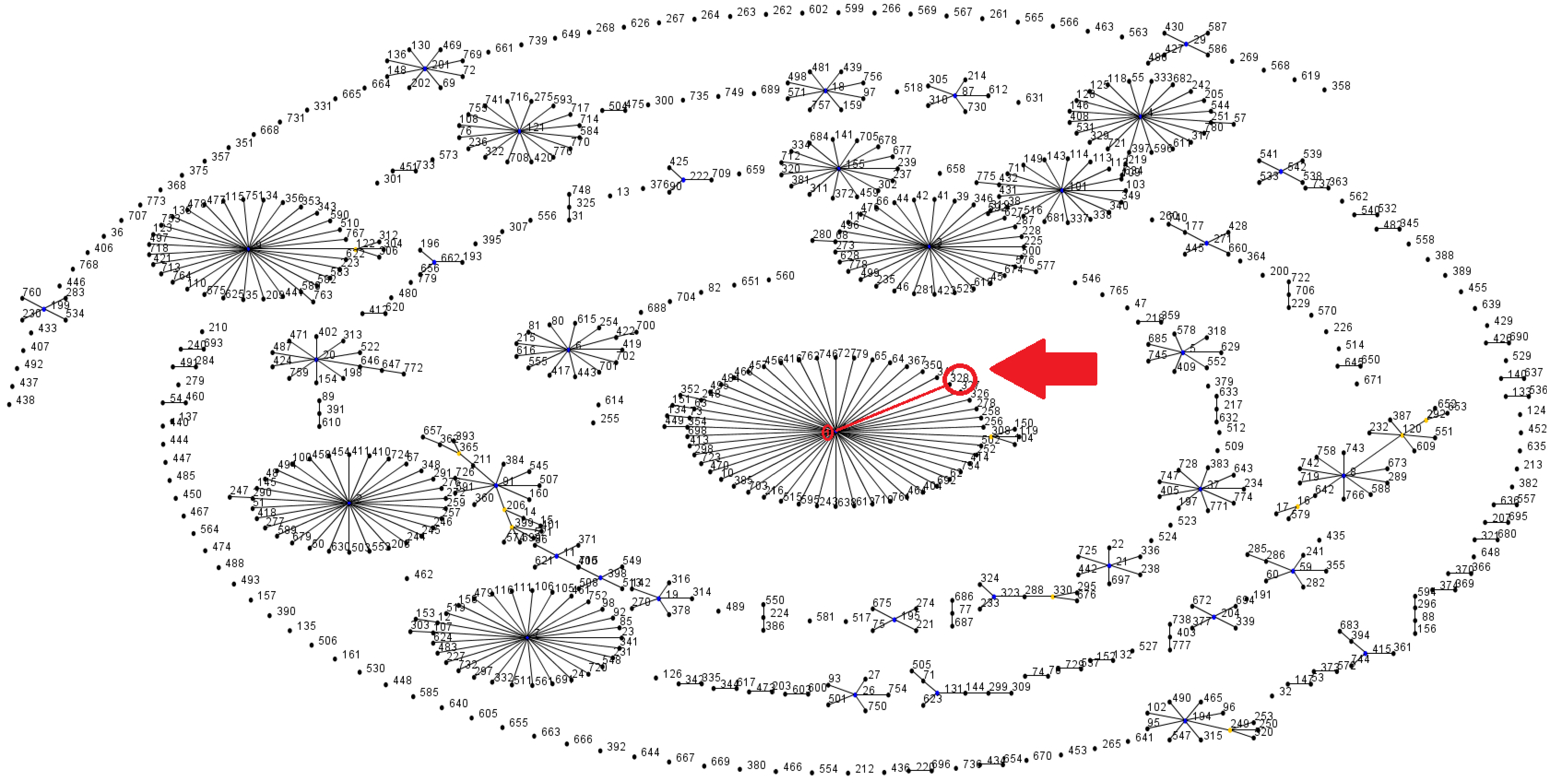

Supplementary Figure S6. Portrait of the L. monocytogenes population structure based on the MLST types. All of the known MLST types for L. monocytogenes (http://bigsdb.web.pasteur.fr/listeria/listeria.html) were considered and analyzed with eBURST software (http://eburst.mlst.net/). The clonal L. monocytogenes serotype 4b strains of India (MLST sequence type 328, 1) among the known L. monocytogenes population are marked with red. The MLST type 328 emerged from MLST ST1 (epidemic clone I) via a single locus variation.
