# Supplementary material for: Presence of a widely disseminated Listeria monocytogenes serotype 4b clone in India
Source: Emerg Microbes Infect. 2016 Jun 8;5(6):e55–. doi: 10.1038/emi.2016.55 (PMC4932648; doi:10.1038/emi.2016.55)
Supplement: Supplementary Figure 7 [file emi201655x8.pdf]

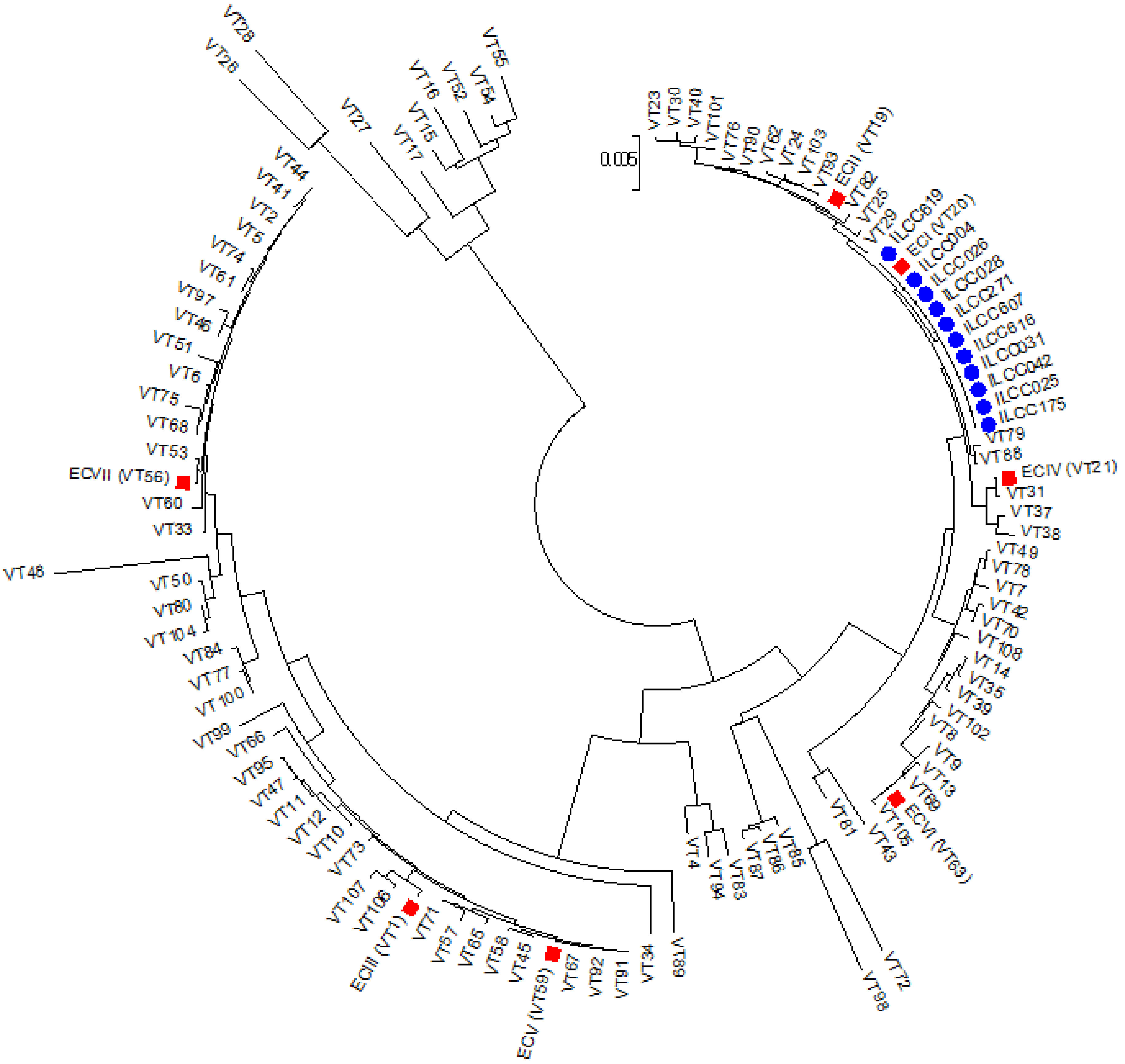

Supplementary Figure 7. An MVLST-based dendrogram illustrating the placement of the 11 studied L. monocytogenes 4b serotype strains. All 11 strains were identified as MVLST VT20, which has also been known as epidemic clone I. (The red solid squares represent the virulence type of the known epidemic clone.) The nucleotide sequences from the segments of the virulence genes[1] were concatenated and aligned, and a neighbor-joining tree was constructed with MEGA 6.0. [2]

1. Zhang W, Jayarao BM, Knabel SJ. Multi-Virulence-Locus Sequence Typing of Listeria monocytogenes. Appl Environ Microbiol 2004; 70: 913–920.

2. Tamura K, Stecher G, Peterson D, Filipski A, Kumar S. MEGA6: Molecular Evolutionary Genetics Analysis version 6.0. Mol Biol Evol 2013; 30: 2725–2729.
