# Supplementary material for: Presence of a widely disseminated Listeria monocytogenes serotype 4b clone in India
Source: Emerg Microbes Infect. 2016 Jun 8;5(6):e55–. doi: 10.1038/emi.2016.55 (PMC4932648; doi:10.1038/emi.2016.55)
Supplement: Supplementary Table 1 [file emi201655x9.pdf]

ILCC Ids	Serotypes	Source	Place	Year	Other info/Ref. if any
ILCC001	4b	Food/Meat/Sausages	Goa	2006	Doijad 2010
ILCC003	4b	Animal/Buffalo/Aborted material	Kolhapur	2001	
***ILCC004**	4b	Animal/Buffalo/Aborted material	Kolhapur	2001	
ILCC005	1/2a	Animal/Animal/Aborted material	Goa	2001	
ILCC006	4b	Animal/Buffalo/Aborted material	Kolhapur	2001	
ILCC007	4b	Food/Fresh water fish/Fish	Nagpur	2007	Jallewar 2007
ILCC008	4b	Food/Fresh water fish/Fish	Nagpur	2007	Jallewar 2007
ILCC010	4b	Food/Fresh water fish/Fish	Nagpur	2007	Jallewar 2007
ILCC011	4b	Food/Fresh water fish/Fish	Nagpur	2007	Jallewar 2007
ILCC012	4b	Food/Fresh water fish/Fish	Nagpur	2007	Jallewar 2007
ILCC013	4b	Food/Fresh water fish/Fish	Nagpur	2007	Jallewar 2007
ILCC014	4b	Food/Fresh water fish/Fish	Nagpur	2007	Jallewar 2007
ILCC015	4b	Animal/Buffalo/Aborted material	Kolhapur	2001	
ILCC016	4b	Animal/Animal/Aborted material	Izzatnagar	2006	Shakuntala 2006
ILCC017	4b	Human/NA/Aborted material	Kolhapur	2009	Kalekar 2011
ILCC022	4b	Animal/Animal/Aborted material	Izzatnagar	2001	Shakuntala 2006
ILCC023	1/2a	Food/Vegetables/Vegetables	Nagpur	2008	
ILCC024	4b	Food/Vegetables/Vegetables	Nagpur	2008	
***ILCC025**	4b	Animal/Jackal/droppings	Nagpur	2006	Kalorey 2006
***ILCC026**	4b	Human/NA/Urine	Goa	2006	Kalekar 2011
ILCC027	1/2b	Human/NA/Blood	Goa	2006	Kalekar 2011
***ILCC028**	4b	Human/NA/Blood	Agra	2006	Kalekar 2011
ILCC029	1/2b	Human/NA/Blood	Pondicherry	2006	Kalekar 2011
ILCC030	4b	Human/NA/Aborted material	Pondicherry	2006	
***ILCC031**	4b	Human/NA/Aborted material	Pondicherry	2006	
ILCC032	4b	Human/NA/Aborted material	Pondicherry	2006	Kalekar 2011
ILCC033	4b	Human/NA/Aborted material	Pondicherry	2006	Kalekar 2011
ILCC035	4b	Human/NA/Aborted material	Goa	2009	Kalekar 2011
ILCC036	4b	Human/NA/Aborted material	Mumbai	2005	Kalekar 2011
ILCC037	4b	Human/NA/Aborted material	Mumbai	2005	Kalekar 2011
ILCC038	4b	Human/NA/Aborted material	Mumbai	2005	Kalekar 2011
ILCC039	4b	Human/NA/Aborted material	Mumbai	2005	Kalekar 2011
ILCC040	1/2b	Animal/Buffalo/Aborted material	Kolhapur	2001	
ILCC041	1/2a	Animal/Goat/Aborted material	Mumbai	2005	
ILCC041a	1/2a	Animal/Animal/Animal	Mumbai	2005	
ILCC041b	4b	Animal/Buffalo/Aborted material	Kolhapur	2001	
***ILCC042**	4b	Animal/Animal/vaginal swab	Izzatnagar	2006	Shakuntala 2006
ILCC043	4b	Animal/Animal/Aborted material	Izzatnagar	2006	Shakuntala 2006
ILCC044	4b	Animal/Animal/Aborted material	Izzatnagar	2006	Shakuntala 2006
ILCC045	4b	Animal/Animal/Animal	Punjab	2007	
ILCC046	4b	Animal/Goat/Aborted material	Mumbai	2005	
ILCC047	4b	Animal/Sheep-healthy/Vaginal-swab	Mumbai	2010	Raorane 2014
ILCC048	4b	Animal/Sheep-healthy/Fecal	Mumbai	2010	Raorane 2014
ILCC049	4b	Animal/Sheep-healthy/blood	Mumbai	2010	Raorane 2014
ILCC050	4b	Animal/Goat-healthy/Vaginal-swab	Mumbai	2010	Raorane 2014
ILCC051	4b	Animal/Animal/Animal	Goa	2002	
ILCC052	1/2b	Animal/Animal/Animal	Kolhapur	2002	
ILCC066	1/2a	Food/Milk/Milk	Mumbai	2004	
ILCC067	1/2a	Food/Milk/Milk	Mumbai	2004	
ILCC079	4b	Human/NA/Aborted material	Goa	2010	Kalekar 2011
ILCC094	4b	Human/NA/Aborted material	Pondicherry	2006	Kalekar 2011
ILCC094a	4b	Human/NA/Aborted material	Pondicherry	2006	Kalekar 2011
ILCC095	1/2b	Human/Human/Human	Delhi	2012	
ILCC097	1/2b	Human/Human/Human	Delhi	2012	
ILCC098	4b	Human/NA/Blood	Agra	2006	Kalekar 2011
ILCC099	1/2b	Human/NA/Blood	Pondicherry	2006	
ILCC115	4b	Animal/Animal/Aborted material	Izzatnagar	2006	Shakuntala 2006

ILCC140	1/2b	Human/NA/Aborted material	Kolhapur	2006	Kalekar 2011
ILCC141	4b	Human/NA/Blood	Goa	2009	Kalekar 2011
ILCC142	4b	Human/NA/Aborted material	Mumbai	2005	Kalekar 2011
ILCC143	4b	Animal/Animal/Aborted material	Izzatnagar	2006	Shakuntala 2006
ILCC144	1/2a	Animal/Buffalo/Aborted material	Goa	2001	
ILCC145	1/2a	Animal/Goat/Aborted material	Mumbai	2005	
ILCC146	4b	Animal/Goat/Aborted material	Mumbai	2005	
ILCC147	4b	Animal/Animal/Animal	Mumbai	2005	
ILCC148	1/2a	Animal/Animal/Animal	Mumbai	2005	
ILCC149	1/2a	Animal/Animal/Animal	Mumbai	2005	
ILCC150	1/2a	Animal/Animal/Animal	Mumbai	2005	
ILCC151	1/2a	Food/Meat/Meat	Mumbai	2004	
ILCC152	1/2a	Food/Meat/Meat	Mumbai	2004	
ILCC153	1/2a	Animal/Animal/Animal	Mumbai	2005	
ILCC154	4b	Food/Meat/Beef	Goa	2006	Doijad 2010
ILCC156	4b	Animal/Animal/a	Goa	2006	Doijad 2010
ILCC158	4b	Food/Meat/Beef	Goa	2006	Doijad 2010
ILCC159	1/2a	Food/Meat/Beef	Goa	2006	Doijad 2010
ILCC160	1/2a	Food/Meat/Beef	Goa	2006	Doijad 2010
ILCC161	4b	Food/Meat/Beef	Goa	2006	Doijad 2010
ILCC164	1/2a	Food/Meat/Sausages	Goa	2006	Doijad 2010
ILCC165	4b	Animal/Buffalo/Aborted material	Kolhapur	2001	
ILCC165a	1/2a	Food/Meat/Sausages	Goa	2006	Doijad 2010
ILCC166	1/2a	Food/Meat/Beef	Goa	2006	Doijad 2010
ILCC171	4b	Animal/India Fox/droppings	Nagpur	2006	Kalorey 2006
ILCC172	4b	Animal/Bison/droppings	Nagpur	2006	Kalorey 2006
ILCC173	4b	Animal/Sambar/droppings	Nagpur	2006	Kalorey 2006
ILCC174	4b	Animal/Wolf/droppings	Nagpur	2006	Kalorey 2006
***ILCC175**	4b	Animal/Sheep-healthy/clinical	Gujarat	2009	Yadava and Roy 2009
ILCC175a	4b	Environmental/Poultry/Poultry	Mumbai	2002	
ILCC176	4b	Environmental/Poultry/Poultry	Mumbai	2002	
ILCC177	4b	Animal/Sheep-healthy/clinical	Gujarat	2009	Yadava and Roy 2009
ILCC177a	4b	Environmental/Poultry/Poultry	Mumbai	2002	
ILCC178	4b	Environmental/Poultry/Poultry	Mumbai	2002	
ILCC179	4b	Environmental/Poultry/Poultry	Mumbai	2002	
ILCC180	4b	Human/NA/Blood	Goa	2009	Kalekar 2011
ILCC180a	4b	Environmental/Poultry/Poultry	Mumbai	2002	
ILCC181	4b	Animal/Sheep-healthy/clinical	Gujarat	2009	Yadava and Roy 2009
ILCC181a	4b	Environmental/Poultry/Poultry	Mumbai	2002	
ILCC182	4b	Animal/Sheep-healthy/clinical	Gujarat	2009	Yadava and Roy 2009
ILCC182a	4b	Environmental/Poultry/Poultry	Mumbai	2002	
ILCC183	4b	Environmental/Poultry/Poultry	Mumbai	2002	
ILCC185	1/2a	Food/Vegetables/Vegetables	Nagpur	2008	
ILCC186	4b	Food/Vegetables/Vegetables	Nagpur	2008	
ILCC187	4b	Food/Vegetables/Vegetables	Nagpur	2008	
ILCC188	4b	Food/Vegetables/Vegetables	Nagpur	2008	
ILCC190	4b	Food/Vegetables/Vegetables	Nagpur	2008	
ILCC191	4b	Food/Vegetables/Vegetables	Nagpur	2008	
ILCC192	1/2a	Food/Vegetables/Vegetables	Nagpur	2008	
ILCC193	4b	Food/Vegetables/Vegetables	Nagpur	2008	
ILCC194	4b	Food/Vegetables/Vegetables	Nagpur	2008	
ILCC195	4b	Food/Vegetables/Vegetables	Nagpur	2008	
ILCC196	1/2b	Food/Seafood /Seafood	Goa	2005	Gawade 2010
ILCC197	1/2b	Food/Seafood /Seafood	Goa	2005	Gawade 2010
ILCC198	1/2a	Food/Seafood /Seafood	Goa	2008	Parihar 2008
ILCC200	1/2a	Food/Seafood /Seafood	Goa	2008	Parihar 2008
ILCC201	1/2a	Food/Seafood /Seafood	Goa	2008	Parihar 2008
ILCC203	1/2a	Food/Seafood /Seafood	Goa	2008	Parihar 2008
ILCC207	1/2a	Food/Seafood /Seafood	Goa	2008	Parihar 2008
ILCC209	1/2a	Food/Seafood /Seafood	Goa	2008	Parihar 2008
ILCC210	1/2a	Food/Seafood /Seafood	Goa	2008	Parihar 2008
ILCC211	1/2a	Food/Seafood /Seafood	Goa	2008	Parihar 2008
ILCC212	1/2a	Food/Seafood /Seafood	Goa	2008	Parihar 2008
ILCC214	1/2a	Food/Seafood /Seafood	Goa	2008	Parihar 2008
ILCC234	4b	Food/Fresh water fish/Fish	Nagpur	2007	Jallewar 2007

ILCC235	4b	Food/Fresh water fish/Fish	Nagpur	2007	Jallewar 2007
ILCC237	1/2a	Human/Human/Human	Nagpur	2010	
ILCC243	4b	Animal/Sheep-healthy/clinical	Gujarat	2009	Yadava and Roy 2009
ILCC244	4b	Human/NA/Aborted material	Mumbai	2005	
ILCC245	4b	Human/Human/Human		2005	
ILCC249	4b	Food/Milk/Raw-milk	Nagpur	2008	Kalorey 2008
ILCC250	1/2a	Food/Milk/Raw-milk	Nagpur	2008	Kalorey 2008
ILCC255	4b	Food/Milk/Raw-milk	Nagpur	2008	Kalorey 2008
ILCC261	4b	Food/Milk/Raw-milk	Nagpur	2008	Kalorey 2008
ILCC262	4b	Food/Milk/Raw-milk	Nagpur	2008	Kalorey 2008
ILCC263	4b	Food/Milk/Raw-milk	Nagpur	2008	Kalorey 2008
ILCC264	4b	Food/Milk/Raw-milk	Nagpur	2008	Kalorey 2008
ILCC265	4b	Food/Milk/Raw-milk	Nagpur	2008	Kalorey 2008
ILCC266	4b	Food/Milk/Raw-milk	Nagpur	2008	Kalorey 2008
ILCC267	4b	Food/Milk/Raw-milk	Nagpur	2008	Kalorey 2008
ILCC268	4b	Food/Milk/Raw-milk	Nagpur	2008	Kalorey 2008
ILCC269	4b	Food/Milk/Raw-milk	Nagpur	2008	Kalorey 2008
ILCC270	4b	Food/Milk/Raw-milk	Nagpur	2008	Kalorey 2008
***ILCC271**	4b	Food/Milk/Raw-milk	Nagpur	2008	Kalorey 2008
ILCC272	4b	Food/Milk/Raw-milk	Nagpur	2008	Kalorey 2008
ILCC273	4b	Food/Milk/Raw-milk	Nagpur	2008	Kalorey 2008
ILCC274	4b	Food/Milk/Raw-milk	Nagpur	2008	Kalorey 2008
ILCC275	4b	Food/Milk/Raw-milk	Nagpur	2008	Kalorey 2008
ILCC276	4b	Animal/Animal/Aborted material	Izzatnagar	2001	Shakuntala 2006
ILCC276a	4b	Food/Milk/Raw-milk	Nagpur	2008	Kalorey 2008
ILCC277	4b	Food/Milk/Raw-milk	Nagpur	2008	Kalorey 2008
ILCC278	4b	Food/Milk/Raw-milk	Nagpur	2008	Kalorey 2008
ILCC279	4b	Food/Milk/Raw-milk	Nagpur	2008	Kalorey 2008
ILCC283	1/2b	Food/Milk/Milk	Mumbai	2004	
ILCC284	1/2a	Food/Milk/Milk	Mumbai	2004	
ILCC285	1/2b	Food/Milk/Milk	Mumbai	2004	
ILCC286	4b	Food/Milk/Milk	Mumbai	2004	
ILCC287	1/2b	Food/Milk/Milk	Mumbai	2004	
ILCC289	1/2b	Food/Milk/Milk	Mumbai	2004	
ILCC291	1/2b	Food/Milk/Milk	Mumbai	2004	
ILCC293	1/2b	Food/Milk/Milk	Mumbai	2004	
ILCC297	1/2b	Food/Milk/Milk	Mumbai	2004	
ILCC298	1/2b	Food/Milk/Milk	Mumbai	2004	
ILCC299	1/2a	Food/Milk/Milk	Mumbai	2004	
ILCC300	1/2b	Food/Milk/Milk	Mumbai	2004	
ILCC301	1/2a	Food/Milk/Milk	Mumbai	2004	
ILCC302	1/2a	Food/Milk/Milk	Mumbai	2004	
ILCC303	1/2a	Food/Milk/Milk	Mumbai	2004	
ILCC304	1/2a	Food/Milk/Milk	Mumbai	2004	
ILCC305	1/2b	Food/Milk/Milk	Mumbai	2004	
ILCC306	1/2a	Food/Milk/Milk	Mumbai	2004	
ILCC307	4b	Food/Milk/Milk	Mumbai	2004	
ILCC309	4b	Food/Milk/Milk	Mumbai	2004	
ILCC310	1/2a	Food/Milk/Milk	Mumbai	2004	
ILCC312	1/2a	Food/Milk/Milk	Mumbai	2004	
ILCC317	1/2a	Food/Milk/Milk	Goa	2007	Parihar 2007
ILCC321	1/2a	Food/Milk/Milk	Goa	2007	Parihar 2007
ILCC324	1/2a	Food/Milk/Milk	Goa	2007	Parihar 2007
ILCC325	1/2a	Food/Milk/Milk	Goa	2007	Parihar 2007
ILCC326	1/2a	Food/Milk/Milk	Goa	2007	Parihar 2007
ILCC327	1/2a	Food/Milk/Milk	Goa	2007	Parihar 2007
ILCC328	1/2a	Food/Milk/Milk	Goa	2007	Parihar 2007
ILCC335	1/2a	Food/Milk/Milk	Goa	2007	Parihar 2007
ILCC336	1/2a	Food/Milk/Milk	Goa	2007	Parihar 2007
ILCC337	1/2a	Food/Milk/Milk	Goa	2007	Parihar 2007
ILCC338	1/2a	Food/Milk/Milk	Goa	2007	Parihar 2007
ILCC343	4b	Food/Milk/Milk	Goa	2007	Parihar 2007
ILCC344	4b	Food/Milk/Milk	Goa	2007	Parihar 2007
ILCC345	4b	Food/Milk/Milk	Goa	2007	Parihar 2007
ILCC346	4b	Food/Milk/Milk	Izzatnagar	2003	

ILCC348	4b	Food/Milk/Milk	Izzatnagar	2003	
ILCC349	4b	Food/Milk/Milk	Izzatnagar	2003	
ILCC350	4b	Food/Milk/Milk	Izzatnagar	2003	
ILCC351	4b	Food/Milk/Milk	Izzatnagar	2003	
ILCC352	4b	Food/Milk/Milk	Izzatnagar	2003	
ILCC353	4b	Food/Milk/Milk	Izzatnagar	2003	
ILCC354	4b	Food/Milk/Milk	Izzatnagar	2003	
ILCC355	4b	Food/Milk/Milk	Izzatnagar	2003	
ILCC357	4b	Food/Milk/Milk	Izzatnagar	2003	
ILCC358	4b	Food/Milk/Milk	Izzatnagar	2003	
ILCC359	4b	Food/Milk/Milk	Izzatnagar	2003	
ILCC360	4b	Food/Milk/Raw-milk	Balkeshwar	2009	Aurora 2009
ILCC361	4b	Food/Milk/Raw-milk	Agra	2009	Aurora 2009
ILCC362	4b	Food/Milk/Milk	Dayalbagh	2009	Aurora 2009
ILCC363	4b	Food/Milk/Milk	Balkeshwar	2009	Aurora 2009
ILCC364	4b	Food/Milk/Milk	Dayalbagh	2009	Aurora 2009
ILCC365	1/2b	Food/Milk/Milk-product	Agra	2009	Aurora 2009
ILCC366	4b	Food/Milk/Milk	Balkeshwar	2009	Aurora 2009
ILCC367	1/2a	Food/Milk/Milk	Agra	2009	Aurora 2009
ILCC368	4b	Food/Milk/Milk	Balkeshwar	2009	Aurora 2009
ILCC369	4b	Food/Milk/Milk	Dayalbagh	2009	Aurora 2009
ILCC370	4b	Food/Milk/Milk	Balkeshwar	2009	Aurora 2009
ILCC371	1/2b	Food/Milk/Milk	Shahganj	2009	Aurora 2009
ILCC372	4b	Food/Milk/Milk-product	Dayalbagh	2009	Aurora 2009
ILCC373	1/2a	Environmental/Dairy-industry/NA	Kolhapur	2010	Doijad 2011
ILCC374	1/2a	Environmental/Dairy-industry/NA	Kolhapur	2010	Doijad 2011
ILCC375	1/2a	Environmental/Dairy-industry/NA	Kolhapur	2010	Doijad 2011
ILCC376	1/2a	Environmental/Dairy-industry/NA	Kolhapur	2010	Doijad 2011
ILCC377	1/2a	Environmental/Dairy-industry/NA	Kolhapur	2010	Doijad 2011
ILCC378	1/2a	Environmental/Dairy-industry/NA	Kolhapur	2010	Doijad 2011
ILCC379	1/2a	Environmental/Dairy-industry/NA	Kolhapur	2010	Doijad 2011
ILCC380	1/2a	Environmental/Dairy-industry/NA	Kolhapur	2010	Doijad 2011
ILCC386	4b	Food/Milk/Milk	Kolhapur	2001	
ILCC395	1/2b	Food/Milk/Milk	Kolhapur	2001	
ILCC397	1/2b	Food/Milk/Raw-milk	Goa	2009	Dcosta 2012
ILCC400	1/2a	Food/Milk/Raw-milk	Goa	2009	Dcosta 2012
ILCC401	1/2a	Food/Milk/Raw-milk	Goa	2009	Dcosta 2012
ILCC402	1/2a	Food/Milk/Raw-milk	Goa	2009	Dcosta 2012
ILCC404	4b	Food/Milk/Raw-milk	Goa	2009	Dcosta 2012
ILCC405	1/2a	Food/Milk/Raw-milk	Goa	2009	Dcosta 2012
ILCC407	1/2a	Food/Milk/Raw-milk	Goa	2009	Dcosta 2012
ILCC409	1/2a	Food/Milk/Raw-milk	Goa	2009	Dcosta 2012
ILCC411	4b	Food/Milk/Raw-milk	Goa	2009	Dcosta 2012
ILCC415	1/2b	Food/Milk/Raw-milk	Goa	2009	Dcosta 2012
ILCC416	1/2b	Food/Milk/Raw-milk	Goa	2009	Dcosta 2012
ILCC419	1/2b	Food/Milk/Raw-milk	Goa	2009	Dcosta 2012
ILCC428	1/2a	Food/seafood/Seafood	Goa	2008	Parihar 2008
ILCC436	1/2a	Food/seafood/Seafood	Goa	2008	Parihar 2008
ILCC451	1/2a	Environmental/Seafood industry/NA	Goa	2009	Rodrigues 2014
ILCC462	4b	Food/Meat/Meat	Mumbai	2004	
ILCC466	4b	Food/Meat/Sausages	Goa	2006	Doijad 2010
ILCC467	1/2a	Food/Meat/Beef	Goa	2006	Doijad 2010
ILCC468	4b	Food/Meat/Meat	Izzatnagar	2000	Barbuddhe 2000
ILCC469	4b	Food/Meat/Meat	Izzatnagar	2001	Barbuddhe 2000
ILCC470	4b	Food/Meat/Meat	Izzatnagar	1997	Barbuddhe 2000
ILCC471	4b	Food/Meat/Meat	Izzatnagar	1997	Barbuddhe 2000
ILCC472	4b	Food/Meat/Meat	Izzatnagar	1997	Barbuddhe 2000
ILCC473	4b	Food/Meat/Meat	Izzatnagar	1997	Barbuddhe 2000
ILCC474	4b	Food/Meat/Meat	Izzatnagar	1997	Barbuddhe 2000
ILCC479	4b	Food/Vegetables/Vegetables	Nagpur	2008	
ILCC484	4b	Food/Vegetables/Vegetables	Nagpur	2008	
ILCC485	4b	Food/Vegetables/Vegetables	Nagpur	2008	
ILCC487	4b	Food/Vegetables/Vegetables	Nagpur	2008	
ILCC489	1/2a	Human/Human/Human	Nagpur	2009	
ILCC491	4b	Animal/Goat /blood	Izzatnagar	1997	Barbuddhe 2000

ILCC492	4b	Animal/Animal/Animal	Mumbai	2005	
ILCC493	4b	Animal/Goat/blood	Izzatnagar	1997	Barbuddhe 2000
ILCC494	4b	Animal/Peacock/droppings	Nagpur	2006	Kalorey 2006
ILCC495	4b	Animal/Deer/droppings	Nagpur	2006	Kalorey 2006
ILCC496	4b	Animal/Animal/Aborted material	Izzatnagar	2006	Shakuntala 2006
ILCC496a	4b	Environmental/Poultry/Poultry	Mumbai	2002	
ILCC498	4b	Animal/Animal/Aborted material	Izzatnagar	2006	Shakuntala 2006
ILCC498a	4b	Environmental/Poultry/Poultry	Mumbai	2002	
ILCC499	4b	Animal/Animal/Aborted material	Izzatnagar	2006	Shakuntala 2006
ILCC499a	4b	Environmental/Poultry/Poultry	Mumbai	2002	
ILCC501	4b	Environmental/Poultry/Poultry	Mumbai	2002	
ILCC502	4b	Environmental/Poultry/Poultry	Mumbai	2002	
ILCC503	4b	Environmental/Poultry/Poultry	Mumbai	2002	
ILCC504	4b	Environmental/Poultry/Poultry	Mumbai	2002	
ILCC506	4b	Environmental/Poultry/Poultry	Mumbai	2002	
ILCC508	4b	Environmental/Poultry/Poultry	Mumbai	2002	
ILCC511	4b	Environmental/Poultry/Poultry	Mumbai	2002	
ILCC515	4b	Environmental/Poultry/Poultry	Mumbai	2002	
ILCC516	4b	Environmental/Poultry/Poultry	Mumbai	2002	
ILCC519	1/2a	Environmental/Dairy-industry/NA	Kolhapur	2010	Doijad 2011
ILCC521	1/2a	Environmental/Dairy-industry/NA	Kolhapur	2010	Doijad 2011
ILCC522	1/2b	Environmental/Dairy-industry/NA	Kolhapur	2010	Doijad 2011
ILCC529	1/2a	Environmental/Dairy-industry/NA	Kolhapur	2010	Doijad 2011
ILCC530	1/2a	Environmental/Dairy-industry/NA	Kolhapur	2010	Doijad 2011
ILCC531	1/2a	Environmental/Food industrial environment/Milk	Goa	2007	Parihar 2007
ILCC532	1/2a	Environmental/Food industrial environment/Milk	Goa	2007	Parihar 2007
ILCC535	1/2a	Food/Milk/Raw-milk	Goa	2009	Dcosta 2012
ILCC538	1/2a	Food/Milk/Raw-milk	Goa	2009	Dcosta 2012
ILCC539	1/2a	Food/Milk/Raw-milk	Goa	2009	Dcosta 2012
ILCC540	1/2a	Food/Milk/Raw-milk	Goa	2009	Dcosta 2012
ILCC541	1/2a	Food/Milk/Raw-milk	Goa	2009	Dcosta 2012
ILCC545	1/2a	Food/Milk/Raw-milk	Goa	2009	Dcosta 2012
ILCC547	1/2a	Food/Milk/Raw-milk	Goa	2009	Dcosta 2012
ILCC553	1/2b	Food/Milk/Goat	Mumbai	2010	Raorane 2014
ILCC557	4b	Human/NA/Aborted material	Mumbai	2005	Kalekar 2011
ILCC557a	4b	Animal/Sheep-healthy/Vaginal-swab	Mumbai	2010	Raorane 2014
ILCC558	1/2b	Animal/Sheep-healthy/Fecal	Mumbai	2010	Raorane 2014
ILCC559	1/2a	Human/Human/Human	Nagpur	2013	
ILCC559a	1/2b	Animal/Goat-healthy/Vaginal-swab	Mumbai	2010	Raorane 2014
ILCC562	4b	Human/NA/Aborted material	Mumbai	2005	Kalekar 2011
ILCC562a	4b	Environmental/Animal rearing environment/Fish	Kankavli	2011	Raorane 2014
ILCC563	1/2b	Animal/Sheep-healthy/?	Mumbai	2010	Raorane 2014
ILCC564	4b	Human/NA/Aborted material	Mumbai	2005	Kalekar 2011
ILCC564a	4b	Animal/Sheep-healthy/Fecal	Mumbai	2010	Raorane 2014
ILCC567	4b	Human/NA/Aborted material	Mumbai	2005	Kalekar 2011
ILCC567a	4b	Animal/Sheep-healthy/blood	Mumbai	2010	Raorane 2014
ILCC568	4b	Animal/Goat-healthy/Vaginal-swab	Mumbai	2010	Raorane 2014
ILCC569	1/2a	Human/Human/Human	Nagpur	2013	
ILCC569a	1/2b	Human/Human/Human	Mumbai	2010	Raorane 2014
ILCC571	4b	Food/Milk/Milk	Goa	2007	Parihar 2007
ILCC584	1/2b	Food/Milk related product/Milk	Shahganj	2009	Aurora 2009
ILCC601	4b	Food/Milk related product/Pedha	Mathura	2007	
ILCC602	4b	Food/Milk related product/Pedha	Mathura	2007	
ILCC603	4b	Food/Milk related product/Pedha	Mathura	2007	
ILCC605	4b	Food/Milk related product/Pedha	Mathura	2007	
***ILCC607**	4b	Invertebrates/Mosquito/Mosquito	Goa	2012	
***ILCC616**	4b	Human/NA/Aborted material	Kolhapur	2007	Kalekar 2011
ILCC617	1/2a	Food/Seafood/Seafood	Goa	2008	Parihar 2008
ILCC618	4b	Human/NA/Blood	Mumbai	2013	
***ILCC619**	4b	Human/NA/CSF	Mumbai	2013	
ILCC620	1/2b	Human/Human/Human	Delhi	2012	
ILCC621	1/2b	Human/Human/Human	Delhi	2012	
ILCC622	1/2b	Human/NA/Aborted material	Mumbai	2013	

ILCC623	1/2b	Human/NA/Blood	Mumbai	2013	
ILCC624	4b	Human/NA/Blood	Mumbai	2013	
ILCC625	4b	Human/Human/Human	Nagpur	2013	
ILCC626	4b	Human/Human/Human	Nagpur	2013	
ILCC627	4b	Human/Human/Human	Nagpur	2013	
ILCC628	1/2a	Human/Human/Human	Nagpur	2013	
ILCC629	1/2a	Human/Human/Human	Nagpur	2013	
ILCC630	1/2a	Human/Human/Human	Nagpur	2013	
ILCC631	1/2a	Human/Human/Human	Nagpur	2013	
ILCC632	1/2a	Human/Human/Human	Nagpur	2013	
ILCC633	1/2a	Human/Human/Human	Nagpur	2013	
ILCC634	4b	Food/Vegetables/NA	Goa	2013	
ILCC635	4b	Food/Seafood/NA	Goa	2013	
ILCC636	4b	Food/Meat/Chevon	Bareilly	2013	
ILCC637	4b	Food/Milk/RTE-milk	Bareilly	2013	
ILCC638	4b	Food/Seafood/NA	Goa	2013	
ILCC639	4b	Food/Vegetables/NA	Bareilly	2013	
ILCC640	4b	Food/Meat/Chicken	Bareilly	2013	
ILCC641	4b	Food/Meat/Chicken	Bareilly	2013	
ILCC642	4b	Food/Meat/Chicken	Bareilly	2013	
ILCC643	1/2b	Food/Milk/RTE-milk	Agra	2013	
ILCC644	4b	Food/Milk related product/Paneer	Agra	2013	
ILCC645	4b	Food/Milk related product/Kulfi (Icecream)	Agra	2013	
ILCC646	1/2a	Food/Milk related product/Paneer	Agra	2013	
ILCC647	4b	Animal/Sheep/Aborted material	Kashmir	2013	
ILCC648	4b	Animal/Sheep/Aborted material	Kashmir	2013	
ILCC649	4b	Animal/Goat/Aborted material	Bareilly	2013	
ILCC650	4b	Animal/Buffalo/Aborted material	Bareilly	2013	
ILCC651	4b	Animal/Sheep/Aborted material	Kashmir	2013	
ILCC652	4b	Animal/Buffalo/Udder-teat-swab	Bareilly	2013	
ILCC653	4b	Animal/Goat/Aborted material	Bareilly	2013	
ILCC654	4b	Animal/Goat/Aborted material	Bareilly	2013	
ILCC655	4b	Animal/Goat/Aborted material	Bareilly	2013	
ILCC656	4b	Animal/Buffalo/Udder-teat-swab	Bareilly	2013	
ILCC657	4b	Animal/Goat/Aborted material	Bareilly	2013	
ILCC658	4b	Animal/Buffalo/Udder-teat-swab	Bareilly	2013	
ILCC659	1/2a	Animal/Buffalo/Aborted material	Nagpur	2013	
ILCC660	4b	Human/NA/Aborted material	Bareilly	2013	
ILCC661	4b	Human/NA/Aborted material	Bareilly	2013	
ILCC662	4b	Human/NA/Aborted material	Bareilly	2013	
ILCC663	4b	Human/NA/Aborted material	Bareilly	2013	
ILCC664	4b	Human/NA/Aborted material	Bareilly	2013	
ILCC665	4b	Human/NA/Aborted material	Bareilly	2013	
ILCC666	4b	Human/NA/Aborted material	Bareilly	2013	
ILCC667	4b	Human/NA/Aborted material	Bareilly	2013	
ILCC668	4b	Human/NA/Aborted material	Bareilly	2013	
ILCC669	4b	Human/NA/Aborted material	Bareilly	2013	
ILCC673	1/2b	Food/Seafood/Fish	Barapani (NE)	2014	
ILCC674	1/2b	Food/Seafood/Fish	Barapani (NE)	2014	
ILCC675	1/2b	Food/Meat/Pork	Barapani (NE)	2014	
ILCC680	1/2a	Food/Meat/Pork	Barapani (NE)	2014	
ILCC683	1/2a	Food/Meat/Chevon	Barapani (NE)	2014	
ILCC691	1/2b	Food//Milk	Barapani (NE)	2014	
ILCC692	1/2b	Food//Milk	Barapani (NE)	2014	
ILCC717	1/2b	Food/Meat/Chevon	Barapani (NE)	2014	
ILCC728	4b	Animal/Sheep-healthy/clinical	Gujarat	2009	Yadava and Roy 2009
ILCC735	4b	Animal/Sheep-healthy/clinical	Gujarat	2009	Yadava and Roy 2009
ILCC736	4b	Animal/Sheep-healthy/clinical	Gujarat	2009	Yadava and Roy 2009
ILCC737	4b	Animal/Sheep-healthy/clinical	Gujarat	2009	Yadava and Roy 2009
ILCC739	4b	Animal/Sheep-healthy/clinical	Gujarat	2009	Yadava and Roy 2009
ILCC745	4b	Animal/Sheep-healthy/clinical	Gujarat	2009	Yadava and Roy 2009
ILCC750	4b	Animal/Sheep-healthy/clinical	Gujarat	2009	Yadava and Roy 2009
ILCC751	4b	Animal/Sheep-healthy/clinical	Gujarat	2009	Yadava and Roy 2009
ILCC752	4b	Animal/Sheep-healthy/clinical	Gujarat	2009	Yadava and Roy 2009

ILCC761	4b	Animal/Sheep-healthy/clinical	Gujarat	2009	Yadava and Roy 2009
ILCC816	4b	Environmental/Mangrove/water-sediment	Goa	2010	Poharkar 2013
ILCC817	1/2a	Food/Meat/Poultry	Shivamoga	2014	
ILCC818	1/2a	Food/Meat/Poultry	Shivamoga	2014	
ILCC819	1/2a	Food/Meat/Poultry	Shivamoga	2014	
ILCC820	4b	Human/Human/Human	Kerla	2014	
ILCC821	1/2a	Food/Meat/Poultry	Shivamoga	2014	
ILCC822	1/2a	Food/Meat/Poultry	Shivamoga	2014	
ILCC823	1/2a	Food/Meat/Poultry	Shivamoga	2014	
ILCC824	1/2a	Food/Meat/Poultry	Shivamoga	2014	
ILCC825	1/2a	Food/Meat/Poultry	Shivamoga	2014	
ILCC826	1/2a	Food/Meat/Poultry	Shivamoga	2014	
ILCC827	4b	Food/Meat/Poultry	Shivamoga	2014	
ILCC828	1/2a	Food/Meat/Poultry	Shivamoga	2014	
ILCC829	1/2a	Food/Meat/Poultry	Shivamoga	2014	
ILCC830	1/2a	Food/Meat/Poultry	Shivamoga	2014	
ILCC831	1/2a	Food/Meat/Poultry	Shivamoga	2014	

Supplementary Table S1. All the isolates were obtained from Indian *Listeria* Culture Collection (ILCC), ICAR Research Complex for Goa, Goa, India. The cultures are now available with Indian *Listeria* Culture Collection, Centre of Excellence and Innovation in Biotechnology on “Translation Centre for Molecular Epidemiology of *Listeria monocytogenes*”. *: Whole genome sequenced strains.
