# Supplementary material for: Presence of a widely disseminated Listeria monocytogenes serotype 4b clone in India
Source: Emerg Microbes Infect. 2016 Jun 8;5(6):e55–. doi: 10.1038/emi.2016.55 (PMC4932648; doi:10.1038/emi.2016.55)
Supplement: Supplementary Table 2 [file emi201655x10.pdf]

Strain	ILCC004	ILCC025	ILCC026	ILCC028	ILCC031	ILCC042	ILCC175	ILCC271	ILCC607	ILCC616	ILCC619
Serotype	4b	4b	4b	4b	4b	4b	4b	4b	4b	4b	4b
Year of isolation	2001	2010	2006	2004	2006	2006	2009	2008	2012	2010	2013
Place of isolation	Kolhapur	Nagpur	Panaji	Agra	Pondicherry	Izzatnagar	Anand	Nagpur	Old Goa	Mumbai	Mumbai
Source	Animal (Abortion)	Animal (Healthy)	Human (Abortion)	Human (Abortion)	Human (Abortion)	Animal (Abortion)	Animal (Healthy)	Milk (Cow)	Invertebrate (Mosquito)	Human (Abortion)	Human (meningitis)
Genome size	2951004	2938001	2950972	2950940	2937927	2939368	2938933	2950867	2950985	2913385	2958462
Genome coverage/ (average fold coverage)	99.01 (106x)	98.52 (57x)	98.95 (236x)	99.17 (173x)	98.72 (75x)	98.79 (79x)	98.78 (76x)	98.21 (220x)	98.94 (224x)	99.05 (122x)	99.26 (173x)
Average read length	207	132	192	210	138	139	133	185	236	203	132
%(G+C)	37.89	37.84	37.88	37.91	37.82	37.83	37.83	37.87	37.88	37.92	37.89
CDS	2935	2873	2927	2926	2930	2882	2870	2928	2929	2870	2950
Prophage	2	2	2	2	0	0	0	2	2	1	1
Prophage size (Kb)	37.852 & 37.649		37.852 & 37.649	37.852 & 37.649				37.852 & 37.649	37.852 & 37.649	37.852	37.852 & 37.649
MLST type	328	328	328	328	328	328	328	328	328	328	1
MVLS Type	VT20	VT20	VT20	VT20	VT20	VT20	VT20	VT20	VT20	VT20	VT20
Accessions number	ERS688179	ERS1065390	ERS688180	ERS688188	ERS1065391	ERS1065392	ERS1065393	ERS688202	ERS688203	ERS688204	ERS1066138

Supplementary Table S2. Details of strains sequenced and their genome features.

*The list of deleted genes is mentioned in supplementary table 3.
