# Supplementary material for: Presence of a widely disseminated Listeria monocytogenes serotype 4b clone in India
Source: Emerg Microbes Infect. 2016 Jun 8;5(6):e55–. doi: 10.1038/emi.2016.55 (PMC4932648; doi:10.1038/emi.2016.55)
Supplement: Supplementary Table 3 [file emi201655x11.pdf]

Annotation	LM F2365 (reference)	ILCC004	ILCC025	ILCC026	ILCC028	ILCC031	ILCC042	ILCC175	ILCC271	ILCC607	ILCC616	ILCC619
1-phosphatidylinositol phosphodiesterase precursor.	F2365_00210	ILCC004_00282	ILCC025_00221	ILCC026_00277	ILCC028_00284	ILCC031_00220	ILCC042_00222	absent	ILCC271_00275	ILCC607_00275	ILCC616_00275	ILCC619_00199
60 kDa chaperonin.	absent	absent	ILCC025_02117	absent	absent	absent	absent	absent	absent	absent	absent	absent
Abi-like protein.	absent	absent	absent	absent	absent	absent	absent	absent	absent	absent	absent	ILCC619_00079
Acetyltransferase (GNAT) family protein.	F2365_00680	ILCC004_00740	absent	ILCC026_00735	ILCC028_00743	ILCC031_00684	absent	ILCC175_00682	ILCC271_00734	ILCC607_00734	ILCC616_00740	ILCC619_00655
Anthrax toxin expression trans-acting positive regulator.	F2365_00491	ILCC004_00554	absent	ILCC026_00549	ILCC028_00556	absent	absent	absent	ILCC271_00547	ILCC607_00547	ILCC616_00554	ILCC619_00471
Aryl-phospho-beta-D-glucosidase BglH.	absent	ILCC004_00021	ILCC025_00006	ILCC026_00021	ILCC028_00021	ILCC031_00006	ILCC042_00006	ILCC175_00006	ILCC271_00021	absent	absent	absent
Beta-glucoside kinase.	absent	absent	ILCC025_00795	absent	absent	absent	absent	absent	absent	absent	absent	absent
bifunctional 3-demethylubiquinone-9 3-methyltransferase/ 2-octaprenyl-6-hydroxy phenol methylase.	F2365_02063	ILCC004_02181	ILCC025_02073	ILCC026_02174	ILCC028_02177	ILCC031_02088	ILCC042_02087	ILCC175_02073	ILCC271_02175	ILCC607_02176	ILCC616_02117	absent
Cystathionine beta-lyase PatB.	F2365_02371	ILCC004_02486	ILCC025_02375	ILCC026_02479	ILCC028_02489	ILCC031_02397	absent	ILCC175_02377	ILCC271_02480	ILCC607_02481	ILCC616_02422	ILCC619_02353
DNA-binding transcriptional activator PspC.	F2365_02488	ILCC004_02603	ILCC025_02496	ILCC026_02596	ILCC028_02607	ILCC031_02519	absent	ILCC175_02496	ILCC271_02597	ILCC607_02598	ILCC616_02539	ILCC619_02531
Exo-glucosaminidase LytG precursor.	F2365_01096	ILCC004_01155	ILCC025_01105	ILCC026_01150	ILCC028_01158	ILCC031_01102	ILCC042_01106	ILCC175_01102	ILCC271_01150	ILCC607_01149	ILCC616_01155	absent
Flagellum site-determining protein YlxH.	F2365_02595	ILCC004_02708	absent	ILCC026_02701	ILCC028_02712	absent	absent	absent	ILCC271_02702	ILCC607_02703	ILCC616_02644	ILCC619_02636
Fumarate hydratase class II.	absent	absent	absent	absent	absent	absent	ILCC042_02304	absent	absent	absent	absent	absent
heat shock protein 90.	absent	absent	absent	absent	absent	absent	absent	absent	absent	absent	absent	ILCC619_00975
HTH-type transcriptional regulator AcrR.	absent	ILCC004_02247	ILCC025_02140	ILCC026_02240	ILCC028_02243	ILCC031_02156	ILCC042_02156	ILCC175_02139	ILCC271_02241	ILCC607_02242	ILCC616_02183	absent
HTH-type transcriptional regulator DegA.	F2365_00040	ILCC004_00040	ILCC025_00026	ILCC026_00040	ILCC028_00040	ILCC031_00025	ILCC042_00026	absent	ILCC271_00040	ILCC607_00040	ILCC616_00040	ILCC619_02955
HTH-type transcriptional repressor GlcR.	F2365_02148	ILCC004_02267	ILCC025_02160	ILCC026_02260	ILCC028_02263	ILCC031_02176	absent	ILCC175_02159	ILCC271_02261	ILCC607_02262	ILCC616_02203	ILCC619_02141
HTH-type transcriptional repressor PurR.	absent	absent	absent	absent	absent	absent	absent	ILCC175_00025	absent	absent	absent	absent
hypothetical protein.	absent	absent	absent	absent	absent	absent	absent	ILCC175_01950	absent	absent	absent	absent
hypothetical protein.	absent	absent	absent	absent	absent	absent	ILCC042_02144	absent	absent	absent	absent	absent
hypothetical protein.	absent	absent	absent	absent	absent	absent	ILCC042_02739	absent	absent	absent	absent	absent
hypothetical protein.	absent	absent	absent	absent	absent	ILCC031_01584	absent	absent	absent	absent	absent	absent
hypothetical protein.	absent	absent	absent	absent	absent	absent	absent	absent	absent	absent	absent	ILCC619_00005
hypothetical protein.	absent	absent	absent	absent	absent	absent	absent	absent	absent	absent	ILCC616_00155	absent
hypothetical protein.	absent	absent	absent	absent	ILCC028_00043	absent	absent	absent	absent	absent	absent	absent

hypothetical protein.	absent	ILCC004_02756	absent	absent	absent	absent	absent	absent	absent	absent	absent	absent
hypothetical protein.	F2365_00076	absent	absent	absent	absent	absent	absent	absent	absent	absent	absent	ILCC619_00002
hypothetical protein.	F2365_00077	absent	absent	absent	absent	absent	absent	absent	absent	absent	absent	ILCC619_00003
hypothetical protein.	F2365_00078	absent	absent	absent	absent	absent	absent	absent	absent	absent	absent	ILCC619_00004
hypothetical protein.	F2365_00079	absent	absent	absent	absent	absent	absent	absent	absent	absent	absent	ILCC619_00006
hypothetical protein.	F2365_00494	absent	absent	absent	ILCC028_00558	absent	absent	absent	absent	ILCC607_00549	absent	absent
hypothetical protein.	F2365_00495	absent	absent	absent	absent	absent	absent	absent	absent	absent	absent	absent
hypothetical protein.	F2365_01264	ILCC004_01386	ILCC025_01276	absent	ILCC028_01383	ILCC031_01286	ILCC042_01292	ILCC175_01289	absent	absent	ILCC616_01323	ILCC619_01274
hypothetical protein.	F2365_01833	absent	ILCC025_01845	absent	absent	ILCC031_01857	ILCC042_01861	ILCC175_01848	absent	absent	absent	ILCC619_01834
hypothetical protein.	F2365_02163	absent	ILCC025_02175	absent	absent	absent	absent	absent	absent	absent	absent	absent
hypothetical protein.	F2365_02292	absent	absent	absent	absent	absent	absent	absent	absent	absent	absent	absent
hypothetical protein.	absent	absent	absent	absent	absent	absent	absent	absent	ILCC271_00739	absent	absent	absent
hypothetical protein.	absent	ILCC004_00163	ILCC025_00098	ILCC026_00158	ILCC028_00165	ILCC031_00097	ILCC042_00099	ILCC175_00098	ILCC271_00156	ILCC607_00156	ILCC616_00156	ILCC619_00080
hypothetical protein.	absent	ILCC004_00164	ILCC025_00099	ILCC026_00159	ILCC028_00166	ILCC031_00098	ILCC042_00100	ILCC175_00099	ILCC271_00157	ILCC607_00157	ILCC616_00157	ILCC619_00081
hypothetical protein.	F2365_00298	ILCC004_00361	absent	ILCC026_00356	ILCC028_00363	ILCC031_00301	ILCC042_00302	ILCC175_00300	ILCC271_00354	ILCC607_00354	ILCC616_00361	ILCC619_00277
hypothetical protein.	F2365_00496	ILCC004_00556	absent	ILCC026_00551	ILCC028_00559	absent	absent	absent	ILCC271_02173	ILCC607_00550	ILCC616_00556	absent
hypothetical protein.	F2365_00603	ILCC004_00663	absent	ILCC026_00658	ILCC028_00666	ILCC031_00607	ILCC042_00603	ILCC175_00603	ILCC271_00657	ILCC607_00657	ILCC616_00663	ILCC619_00578
hypothetical protein.	F2365_01048	ILCC004_01107	ILCC025_01056	ILCC026_01102	ILCC028_01110	ILCC031_01054	ILCC042_01057	absent	ILCC271_01102	ILCC607_01101	ILCC616_01107	ILCC619_01059
hypothetical protein.	F2365_01354	ILCC004_01477	absent	ILCC026_01471	ILCC028_01474	ILCC031_01382	ILCC042_01387	ILCC175_01381	ILCC271_01471	ILCC607_01470	ILCC616_01414	ILCC619_01364
hypothetical protein.	F2365_01834	ILCC004_01956	absent	ILCC026_01949	ILCC028_01952	absent	absent	absent	ILCC271_01950	ILCC607_01948	ILCC616_01892	absent
hypothetical protein.	F2365_02034	ILCC004_02156	ILCC025_02049	ILCC026_02149	ILCC028_02152	ILCC031_02063	absent	ILCC175_02051	ILCC271_02150	ILCC607_02148	ILCC616_02092	ILCC619_02034
hypothetical protein.	F2365_02180	ILCC004_02298	absent	ILCC026_02291	ILCC028_02294	ILCC031_02207	ILCC042_02207	ILCC175_02190	ILCC271_02292	ILCC607_02293	ILCC616_02234	ILCC619_02172
hypothetical protein.	F2365_02659	ILCC004_02773	ILCC025_02665	ILCC026_02765	ILCC028_02776	ILCC031_02688	ILCC042_02678	absent	ILCC271_02766	ILCC607_02767	ILCC616_02708	ILCC619_02700
hypothetical protein.	F2365_02720	ILCC004_02834	ILCC025_02727	ILCC026_02826	ILCC028_02837	ILCC031_02750	absent	ILCC175_02726	ILCC271_02827	ILCC607_02828	ILCC616_02769	ILCC619_02760
hypothetical protein.	F2365_02798	ILCC004_02912	ILCC025_02810	ILCC026_02904	ILCC028_02915	absent	ILCC042_02818	ILCC175_02806	ILCC271_02905	ILCC607_02906	ILCC616_02847	ILCC619_02838
hypothetical protein. NOTE: GHMP kinases C terminal.	absent	absent	absent	absent	absent	ILCC031_02925	absent	absent	absent	absent	absent	absent
Internalin B precursor related protein.	absent	absent	absent	absent	absent	absent	absent	absent	absent	absent	absent	ILCC619_01107
Lichenan-specific phosphotransferase enzyme IIB component.	absent	ILCC004_00453	ILCC025_00396	ILCC026_00448	ILCC028_00455	ILCC031_00395	ILCC042_00396	ILCC175_00395	absent	absent	absent	absent
Listeria-Bacteroides repeat domain (List_Bact_rpt).	F2365_00492	ILCC004_00555	absent	ILCC026_00550	ILCC028_00557	absent	absent	absent	ILCC271_00549	ILCC607_00548	ILCC616_00555	absent

Luminescence regulatory protein LuxO.	F2365_01755	absent	absent	absent	absent	absent	absent	absent	absent	absent	absent	absent
Muramidase-2 precursor.	F2365_02701	ILCC004_02815	ILCC025_02708	ILCC026_02807	ILCC028_02818	ILCC031_02731	ILCC042_02720	ILCC175_02707	ILCC271_02808	ILCC607_02809	ILCC616_02750	absent
Penicillin-binding protein 2B.	F2365_02077	ILCC004_02195	ILCC025_02087	ILCC026_02188	ILCC028_02191	ILCC031_02103	absent	absent	ILCC271_02189	ILCC607_02190	ILCC616_02131	ILCC619_02070
PRD domain protein.	absent	absent	absent	absent	absent	absent	ILCC042_00799	absent	absent	absent	absent	absent
Propanediol utilization protein PduA.	absent	ILCC004_01217	ILCC025_01167	ILCC026_01212	ILCC028_01220	ILCC031_01163	ILCC042_01169	ILCC175_01165	ILCC271_01212	ILCC607_01211	ILCC616_01217	ILCC619_01176
putative oxidoreductase YdgJ.	F2365_02207	ILCC004_02325	ILCC025_02219	ILCC026_02318	ILCC028_02321	absent	ILCC042_02234	ILCC175_02217	ILCC271_02319	ILCC607_02320	ILCC616_02261	ILCC619_02199
putative PTS system mannitol-specific transporter subunit IIA.	absent	absent	absent	absent	absent	absent	absent	ILCC175_02682	absent	absent	absent	absent
Putative transposase DNA-binding domain protein.	absent	absent	absent	ILCC026_00156	absent	absent	absent	absent	absent	absent	absent	absent
Replication protein.	absent	absent	absent	ILCC026_00157	absent	absent	absent	absent	absent	absent	absent	absent
RNA polymerase sigma-54 factor 1.	absent	absent	absent	absent	ILCC028_02581	absent	absent	absent	absent	absent	absent	absent
Transposon Tn10 TetC protein.	F2365_02465	ILCC004_02580	absent	ILCC026_02573	ILCC028_02584	ILCC031_02496	ILCC042_02488	ILCC175_02473	ILCC271_02574	ILCC607_02575	ILCC616_02516	ILCC619_02447
tRNA(Ile)-lysidine synthase.	absent	ILCC004_01444	ILCC025_01337	ILCC026_01438	ILCC028_01441	ILCC031_01348	ILCC042_01353	ILCC175_01348	ILCC271_01438	ILCC607_01437	ILCC616_01381	absent

Supplementary Table S3. Total list of genes absent in the sequenced *L. monocytogenes* 4b strains from India as compared to *L. monocytogenes* F2365. Genes that are uniquely present to the strains are yellow highlighted.
