# Supplementary material for: Presence of a widely disseminated Listeria monocytogenes serotype 4b clone in India
Source: Emerg Microbes Infect. 2016 Jun 8;5(6):e55–. doi: 10.1038/emi.2016.55 (PMC4932648; doi:10.1038/emi.2016.55)
Supplement: Supplementary Table 4 [file emi201655x12.pdf]

Reference position F2365	Refernce_nucleotide	Altered_nucleotide	ILCC004	ILCC026	ILCC028	ILCC025	ILCC031	ILCC042	ILCC175	ILCC271	ILCC607	ILCC616	ILCC619	Gene_Product
1771	C	T	0	0	0	0	0	0	0	0	0	0	1	Ktr system potassium uptake protein B
5965	C	A	0	0	0	0	0	0	0	0	0	0	1	DNA replication and repair protein RecF
7796	G	A	1	1	1	1	1	1	1	1	1	1	1	DNA gyrase subunit B
9000	T	C	1	1	1	1	1	1	1	1	1	1	0	DNA gyrase subunit A
10265	G	A	1	0	0	0	0	1	0	0	0	1	0	DNA gyrase subunit A
13046	A	T	0	0	0	0	0	0	0	0	0	0	1	mevalonate kinase
37864	C	T	1	1	1	1	1	1	1	1	1	1	0	hypothetical protein
39056	C	T	0	0	0	0	0	0	0	0	0	0	1	Sugar phosphatase YidA
41853	C	T	0	0	0	0	0	0	0	0	0	0	1	Endoglucanase D precursor
46805	T	A	1	1	1	1	1	1	1	1	1	1	0	intergenic
49661	G	A	0	0	0	0	0	0	0	0	0	0	1	Putative agmatine deiminase
59328	T	A	0	0	0	0	0	0	0	0	0	0	1	Sensor histidine kinase DpiB
60633	C	A	1	1	1	1	1	1	1	1	1	1	0	accessory gene regulator protein A
68512	G	C	0	0	0	0	0	0	0	0	0	0	1	hypothetical protein
90111	C	T	1	1	1	1	1	1	1	1	1	1	0	6-O-methylguanine DNA methyltransferase
93406	G	A	0	0	0	0	0	0	0	0	0	0	1	Ribonuclease
115050	A	G	0	0	0	0	0	0	0	0	1	0	0	PTS system mannose-specific transporter subunit IIAB
126654	C	T	1	1	1	1	1	1	1	1	1	1	1	ABC transporter ATP-binding protein/permease
149373	T	G	0	0	0	0	0	1	0	0	0	0	0	peptide ABC transporter substrate-binding protein
155746	C	T	1	1	1	1	1	1	1	1	1	1	0	hypothetical protein
159466	C	G	1	1	1	1	1	1	1	1	1	1	1	solute-binding family 5 protein
177872	C	G	0	0	0	0	0	0	0	0	0	0	1	hypothetical protein
183215	T	A	0	0	0	0	0	0	0	0	0	1	0	intergenic
183342	T	C	0	0	0	0	0	0	0	0	0	1	0	xylose repressor protein
190770	C	T	0	0	0	0	0	0	0	0	0	0	1	Alpha-xylosidase
191876	G	T	1	1	1	1	1	1	1	1	1	1	0	alpha-glucosidase
202365	A	G	1	1	1	1	1	1	1	1	1	1	1	hypothetical protein
211295	A	G	1	1	1	1	1	1	1	1	1	1	1	listeriolysin O
220056	A	T	1	1	1	1	1	1	1	1	1	1	0	L-lactate dehydrogenase
234757	A	G	0	0	0	0	0	0	0	0	0	0	1	Type III pantothenate kinase
254927	G	A	0	0	0	0	0	0	0	0	0	0	1	Putative ATP:guanido phosphotransferase
266088	C	A	1	0	0	0	0	0	0	0	0	0	0	cysteinyl-tRNA synthetase
266090	T	A	1	0	0	0	0	0	0	0	0	0	0	cysteinyl-tRNA synthetase
266091	T	A	1	0	0	0	0	0	0	0	0	0	0	cysteinyl-tRNA synthetase
267188	T	C	1	1	1	1	1	1	1	1	1	1	1	RNA methyltransferase
301296	C	G	1	1	1	1	1	1	1	1	1	1	0	intergenic
306754	A	G	1	1	1	1	1	1	1	1	1	1	0	transporter
331305	C	A	0	0	0	0	0	0	0	0	0	0	1	Two-component system YycF/YycG regulatory protein YycH
333383	G	T	0	0	0	0	0	0	1	0	0	0	0	serine protease
335614	C	T	1	1	1	1	1	1	1	1	1	1	0	rRNA large subunit methyltransferase
344404	G	A	0	0	0	0	0	0	0	0	0	0	1	intergenic
354068	G	C	1	1	1	1	1	1	1	1	1	1	1	hydroxyethylthiazole kinase
360679	C	T	1	1	1	1	1	1	1	1	1	1	0	hypothetical protein
366287	C	T	1	1	1	1	1	1	1	1	1	1	0	hypothetical protein
369657	G	A	0	0	0	0	0	0	0	0	0	0	1	Internalin-J precursor
372754	G	A	0	0	0	0	0	0	0	0	0	0	1	Internalin-A precursor
408791	T	C	1	1	1	1	1	1	1	1	1	1	1	transcriptional regulator
420412	G	A	0	0	0	0	0	0	0	0	1	0	0	thiamine-pyrophosphate-requiring enzyme
429057	T	C	1	1	1	1	1	1	1	1	1	1	1	intergenic
441789	C	T	0	0	0	0	0	0	0	0	0	1	0	hypothetical protein
456381	C	T	1	1	1	1	1	1	1	1	1	1	1	cof family hydrolase
458429	G	T	0	0	0	0	0	0	0	0	0	0	1	putative licABCH operon regulator
460548	G	A	0	0	0	0	0	0	0	0	0	0	1	Heat-responsive suppressor HrsA

465256	T	C	0	0	0	0	0	0	0	0	0	1	0	intergenic
474274	G	T	0	0	0	0	0	0	0	0	0	0	1	hypothetical protein
486998	T	C	1	1	1	1	1	1	1	1	1	1	1	intergenic
489455	C	A	0	0	0	0	0	0	0	0	0	0	1	intergenic
494387	C	T	0	0	0	0	0	0	0	0	0	0	1	Beta-lactam-inducible penicillin-binding protein
506235	G	A	1	1	1	1	1	1	1	1	1	1	0	cytosine/purines uracil thiamine allantoin permease
533215	C	T	0	0	0	0	0	0	0	0	0	0	1	HTH-type transcriptional regulator GltC
538185	G	A	0	0	0	0	0	0	0	0	0	0	1	HTH-type transcriptional regulator GltC
556886	A	T	1	1	1	1	1	1	1	1	1	1	0	cell wall surface anchor protein
557610	C	T	0	0	0	0	0	0	0	1	0	0	0	universal stress protein
568561	T	C	1	1	1	1	1	1	1	1	1	1	1	MerR family transcriptional regulator
573557	A	G	1	1	1	1	1	1	1	1	1	1	1	glycosyl transferase family protein
576117	T	C	1	1	1	1	1	1	1	1	1	1	0	hypothetical protein
588425	G	A	1	1	1	1	1	1	1	1	1	1	0	PTS system glucitol/sorbitol-specific transporter subunit IIBC
589922	T	C	1	1	1	1	1	1	1	1	1	1	1	hypothetical protein
590107	A	C	1	1	1	1	1	1	1	1	1	1	0	hypothetical protein
595402	C	T	0	0	0	0	0	0	0	0	0	0	1	Internalin-A precursor
596108	T	C	1	1	1	1	1	1	1	1	1	1	1	hypothetical protein
610432	C	A	0	0	0	0	0	0	0	0	0	0	1	Imidazoleglycerol-phosphate dehydratase
614820	C	T	1	1	1	1	1	1	1	1	1	1	0	methylated-DNA--protein-cysteine methyltransferase
614932	C	T	1	1	1	1	1	1	1	1	1	1	0	methylated-DNA--protein-cysteine methyltransferase
618597	C	T	0	0	0	0	0	0	0	0	0	0	1	6-phospho-beta-glucosidase GmuD
620209	A	T	1	1	1	1	1	1	1	1	1	1	0	intergenic
621035	C	A	0	0	0	0	0	0	0	0	0	0	1	Internalin-J precursor
625098	T	C	1	1	1	1	1	1	1	1	1	1	1	hypothetical protein
632469	G	A	0	0	0	0	0	0	0	0	0	0	1	hypothetical protein
635084	G	A	0	0	0	0	0	0	0	0	0	0	1	hypothetical protein
637750	G	A	0	0	0	0	0	0	0	0	0	0	1	DegV domain-containing protein
650047	T	C	1	1	1	1	1	1	1	1	1	1	1	hypothetical protein
658874	A	G	1	1	1	1	1	1	1	1	1	1	1	FMN-dependent NADH-azoreductase 1
659070	C	T	0	0	0	0	0	0	0	0	0	0	1	intergenic
659538	A	G	0	0	0	0	0	0	0	0	0	0	1	putative HTH-type transcriptional regulator/GBAA_1941/BAS1801
670159	G	A	0	0	0	0	0	0	0	0	0	0	1	hypothetical protein
671234	C	T	0	0	0	0	0	0	0	0	0	0	1	Collagen adhesin precursor
695618	T	C	0	0	0	0	0	0	0	0	0	0	1	Catechol-2,3-dioxygenase
702836	C	T	0	0	0	0	0	0	0	0	0	0	1	intergenic
714068	T	C	0	0	0	0	0	0	0	0	0	0	1	Internalin-J precursor
735005	G	C	0	0	0	0	0	0	0	1	0	0	0	chemotaxis protein MotB
735725	G	A	0	0	0	0	0	0	0	0	1	0	0	chemotaxis protein MotB
737811	G	A	0	0	0	0	0	0	0	1	0	0	0	glycosyl transferase family protein
739442	A	C	0	0	0	0	0	0	1	0	0	0	0	flagellin
739514	G	A	1	0	0	0	0	0	0	0	0	0	0	flagellin
759549	C	G	1	1	1	1	1	1	1	1	1	1	1	flagellar MS-ring protein
769535	C	T	0	0	0	0	0	0	0	0	0	0	1	Methyl-accepting chemotaxis protein 4
770582	T	C	0	0	0	0	0	0	0	0	0	0	1	hypothetical protein
771053	A	G	1	0	0	0	0	0	0	0	0	0	0	intergenic
771267	A	G	1	1	1	1	1	1	1	1	1	1	1	glucosamine--fructose-6-phosphate aminotransferase
791073	G	A	1	1	1	1	1	1	1	1	1	1	0	glyoxalase
808554	C	A	1	1	1	1	1	1	1	1	1	1	0	sigma-54 dependent transcriptional regulator
810704	T	C	1	1	1	1	1	1	1	1	1	1	1	intergenic
818142	T	A	0	0	0	0	0	0	0	0	0	0	1	Internalin-J precursor
820801	G	A	1	1	1	1	1	1	1	1	1	1	1	hypothetical protein
842793	T	A	0	0	0	0	0	0	1	0	0	0	0	hypothetical protein
857593	A	G	0	0	1	0	0	0	0	0	1	0	0	pyruvate flavodoxin/ferredoxin oxidoreductase
861269	C	T	0	0	0	0	0	0	0	0	0	0	1	Fructose-1,6-bisphosphatase class 3
867080	G	A	1	1	1	1	1	1	1	1	1	1	1	intergenic
880511	G	A	0	0	0	0	0	0	0	0	0	0	1	Bacterial Ig-like domain (group 3)
889414	G	A	1	0	0	0	0	1	0	0	0	1	0	amidase
903906	G	C	0	0	0	0	0	0	0	0	1	0	0	phosphoglucomutase/phosphomannomutase

903907	C	T	0	0	0	0	0	0	0	0	1	0	0	phosphoglucomutase/phosphomannomutase
903911	A	T	0	0	0	0	0	0	0	0	1	0	0	phosphoglucomutase/phosphomannomutase
903912	A	T	0	0	0	0	0	0	0	0	1	0	0	phosphoglucomutase/phosphomannomutase
907485	A	T	1	1	1	1	1	1	1	1	1	1	1	intergenic
913176	T	G	0	0	1	0	0	0	0	0	0	0	0	PTS fructose transporter subunit IIA
928246	C	T	0	0	0	0	0	0	1	0	0	0	0	membrane protein
928638	G	A	0	0	0	0	0	0	0	0	0	1	0	anti-sigma B factor RbsT
934289	T	C	1	1	1	1	1	1	1	1	1	1	1	S1 RNA-binding domain-containing protein
937163	G	A	1	1	1	1	1	1	1	1	1	1	0	hypothetical protein
937507	T	C	1	1	1	1	1	1	1	1	1	1	0	hypothetical protein
947058	A	T	1	1	1	1	1	1	1	1	1	1	1	membrane protein
963887	A	G	0	0	0	0	0	0	0	0	0	0	1	Fatty acid metabolism regulator protein
974232	A	G	0	0	0	0	0	0	0	0	0	0	1	Putative tRNA (cytidine(34)-2'-O)-methyltransferase
981089	C	T	0	0	0	0	1	0	0	0	0	0	0	MBL fold protein
981846	A	G	0	0	0	0	0	0	0	0	0	0	1	ComE operon protein 1
984814	G	A	0	0	0	0	0	0	0	0	0	0	1	pheromone autoinducer 2 transporter
986193	T	A	0	0	0	0	0	0	0	0	0	0	1	Alpha/beta hydrolase family protein
992943	G	A	0	0	0	0	0	0	0	0	0	0	1	putative undecaprenyl-phosphate N-acetylglucosaminyl 1-phosphate transferase
998537	C	T	0	0	0	0	0	0	0	0	0	0	1	CYTH domain protein
1006540	G	A	0	0	0	0	0	0	0	0	0	0	1	D-alanine--poly(phosphoribitol) ligase subunit 1
1026229	C	A	1	1	1	1	1	1	1	1	1	1	1	hypothetical protein
1029598	C	T	1	1	1	1	1	1	1	1	1	1	1	ATP-dependent Clp protease, ATP-binding subunit ClpE
1032811	C	A	0	0	0	0	0	0	0	0	0	0	1	zeta-carotene-forming phytoene desaturase
1036563	C	A	0	0	0	0	0	0	0	0	0	0	1	hypothetical protein
1067823	A	G	1	1	1	1	1	1	1	1	1	1	1	hypothetical protein
1078172	A	G	1	1	1	1	1	1	1	1	1	1	1	intergenic
1090259	T	C	1	1	1	1	1	1	1	1	1	1	1	GTP-binding protein TypA
1099622	T	C	0	0	0	0	0	0	0	1	0	0	0	ABC transporter substrate-binding protein
1108883	C	T	0	0	0	0	0	0	0	0	0	0	1	hypothetical protein
1117847	A	G	1	1	1	1	1	1	1	1	1	1	1	teichoic acid biosynthesis domain-containing protein
1129231	T	C	0	0	0	0	0	0	0	0	0	0	1	Nitroreductase family protein
1132280	T	A	1	1	1	1	1	1	1	1	1	1	1	CAAX amino terminal protease
1143825	A	G	0	0	0	0	0	0	0	0	0	0	1	ATP-binding/permease protein CydD
1146508	A	T	0	0	1	0	0	0	0	0	0	0	0	hypothetical protein
1149053	A	T	0	0	0	0	0	0	0	0	0	0	1	intergenic
1149054	G	T	0	0	0	0	0	0	0	0	0	0	1	intergenic
1149736	A	C	1	1	1	1	1	1	1	1	1	1	1	cell wall surface anchor protein
1162100	G	A	0	0	0	0	0	0	0	0	0	0	1	Propanediol dehydratase large subunit
1164320	A	C	1	1	1	1	1	1	1	1	1	1	1	propanediol utilization diol dehydratase reactivation protein
1165843	G	A	0	0	0	0	0	0	0	0	0	0	1	Diol dehydratase-reactivating factor alpha subunit
1169192	G	A	0	0	0	0	0	0	0	0	0	0	1	Carbon dioxide concentrating mechanism protein CcmL
1172730	C	T	0	0	0	0	0	0	0	0	0	0	1	Aldehyde-alcohol dehydrogenase
1176850	T	C	1	1	1	1	1	1	1	1	1	1	0	intergenic
1177045	G	T	1	1	1	1	1	1	1	1	1	1	0	hypothetical protein
1184966	C	T	1	1	1	1	1	1	1	1	1	1	0	ethanolamine utilization protein
1194586	C	A	0	0	0	0	0	0	0	0	0	0	1	Cobyrinic acid A,C-diamide synthase
1209443	G	A	1	1	1	1	1	1	1	1	1	1	0	ATP:cob(I)alamin adenosyltransferase
1215317	C	T	0	0	0	0	0	0	0	0	0	0	1	Putative aminopeptidase YsdC
1226335	A	G	0	0	0	0	0	0	0	0	0	0	1	Multiple antibiotic resistance protein MarR
1252319	G	A	0	0	0	0	0	0	0	0	0	0	1	2,3-bisphosphoglycerate-dependent phosphoglycerate mutase
1256960	T	C	1	1	1	1	1	1	1	1	1	1	1	Transporter
1258101	T	A	1	1	1	1	1	1	1	1	1	1	0	intergenic
1263550	C	T	1	1	1	1	1	1	1	1	1	1	0	intergenic
1278964	C	A	0	0	0	0	0	0	0	0	0	0	1	intergenic
1283173	A	C	0	0	0	0	0	0	0	1	0	0	0	tRNA (uracil-5-)-methyltransferase Gid
1286979	A	G	0	0	0	0	0	0	0	0	0	0	1	GTP-sensing transcriptional pleiotropic repressor CodY
1288773	A	G	1	1	1	1	1	1	1	1	1	1	1	aldose epimerase
1300945	C	G	0	0	0	0	0	0	0	1	0	0	0	intergenic

1336918	A	G	1	1	1	1	1	1	1	1	1	1	1	tRNA pseudouridine synthase B
1350580	A	C	0	0	0	0	0	0	0	0	0	0	1	Type II secretion system protein F
1353322	G	A	0	0	0	0	0	0	0	1	0	0	0	glycine cleavage system aminomethyltransferase T
1387671	C	T	0	0	0	0	0	0	0	0	0	0	1	putative rhodanese-related sulfurtransferase
1405833	T	C	0	0	0	0	0	0	0	0	0	0	1	recombinase A
1410330	C	A	1	1	1	1	1	1	1	1	1	1	0	DNA mismatch repair protein MutS
1412899	T	C	0	0	0	0	0	0	0	0	0	0	1	DNA mismatch repair protein MutL
1417168	T	C	0	0	0	0	0	0	0	0	0	0	1	Formate acetyltransferase
1426302	T	G	0	0	0	0	0	0	0	0	0	0	1	VanZ like family protein
1440367	C	T	1	1	1	1	1	1	1	1	1	1	1	hypothetical protein
1446160	C	A	0	0	0	0	0	0	0	0	0	0	1	Ribonuclease J 2
1446341	A	G	1	0	0	0	0	0	0	0	0	0	0	metallo-beta-lactamase
1446886	T	C	0	0	0	0	0	0	0	0	0	0	1	Ribonuclease J 2
1466219	G	A	0	0	0	0	0	0	0	0	1	0	0	RNA polymerase sigma factor RpoD
1472611	C	T	0	0	0	0	0	0	0	0	0	0	1	Glycine--tRNA ligase beta subunit
1481888	T	G	0	0	0	0	0	0	0	0	1	0	0	intergenic
1510492	G	A	1	1	1	1	1	1	1	1	1	1	0	DedA family protein
1513003	A	T	1	1	1	1	1	1	1	1	1	1	1	alanyl-tRNA synthetase
1525276	C	T	1	1	1	1	1	1	1	1	1	1	0	carbon-sulfur lyase
1528373	A	G	0	0	0	0	0	0	0	0	0	0	1	intergenic
1541494	T	A	0	0	0	0	0	0	0	1	0	0	0	hypothetical protein
1558288	T	C	1	1	1	1	1	1	1	1	1	1	1	intergenic
1591850	G	A	0	0	0	0	0	0	0	0	0	0	1	Acetyl-coenzyme A carboxylase carboxyl transferase subunit alpha
1595740	T	C	1	1	1	1	1	1	1	1	1	1	1	DNA polymerase III subunit alpha
1596431	T	C	0	0	0	0	0	0	0	0	0	0	1	DNA polymerase III subunit alpha
1598254	G	A	1	0	0	0	0	0	0	0	0	0	0	hypothetical protein
1598751	G	C	0	0	0	0	0	0	0	0	1	0	0	hypothetical protein
1602810	G	A	1	1	1	1	1	1	1	1	1	1	0	acetate kinase
1619378	G	T	1	1	1	1	1	1	1	1	1	1	1	30S ribosomal protein S4
1641338	G	A	0	0	0	0	0	0	0	0	0	0	1	Putative dipeptidase
1658812	T	C	0	0	0	0	0	0	0	0	0	0	1	intergenic
1675514	A	G	1	1	1	1	1	1	1	1	1	1	1	hypothetical protein
1679857	G	T	1	1	1	1	1	1	1	1	1	1	0	ABC transporter ATP-binding protein/permease
1687161	G	T	1	1	1	1	1	1	1	1	1	1	0	PTS system sucrose-specific transporter subunit IIBC
1705173	A	C	1	1	1	1	1	1	1	1	1	1	1	hypothetical protein
1709346	C	T	0	0	0	0	0	0	0	0	0	0	1	2-succinyl-6-hydroxy-2, 4-cyclohexadiene-1-carboxylate synthase
1719576	G	A	1	1	1	1	1	1	1	1	1	1	0	5-methyltetrahydropteroyltriglutamate/homocysteine S-methyltransferase
1729010	C	T	0	0	0	0	0	0	0	0	0	1	0	intergenic
1730518	G	A	1	1	1	1	1	1	1	1	1	1	0	D-isomer specific 2-hydroxyacid dehydrogenase
1750544	G	A	0	0	0	0	0	0	0	0	0	0	1	hypothetical protein
1754966	T	C	1	1	1	1	1	1	1	1	1	1	0	MFS transporter
1761565	C	T	0	0	0	0	0	0	0	0	0	0	1	Levansucrase and sucrase synthesis operon antiterminator
1776559	C	T	1	1	1	1	1	1	1	1	1	1	0	ABC transporter substrate-binding protein
1785472	T	A	1	1	1	1	1	1	1	1	1	1	1	GltC family transcriptional regulator
1789238	G	A	1	0	0	0	0	0	0	0	0	0	0	amino acid ABC transporter permease
1789239	T	A	1	0	0	0	0	0	0	0	0	0	0	intergenic
1790669	T	A	0	0	0	0	0	0	0	0	0	0	1	Sensor histidine kinase GraS
1795666	T	A	1	1	1	1	1	1	1	1	1	1	1	ABC transporter permease
1795993	A	C	1	1	1	1	1	1	1	1	1	1	1	ABC transporter permease
1807051	A	T	0	0	0	0	0	0	0	0	0	0	1	CamS sex pheromone cAM373 precursor
1811550	C	T	0	0	0	0	0	0	0	0	0	0	1	ATP-dependent DNA helicase PcrA
1820797	G	C	1	1	1	1	1	1	1	1	1	1	1	phosphoribosylformylglycinamidine synthase II
1821678	G	A	0	0	0	0	1	0	0	0	0	0	0	phosphoribosylformylglycinamidine synthase subunit PurL
1823238	A	G	1	1	1	1	1	1	1	1	1	1	1	phosphoribosylformylglycinamidine synthase I
1824466	A	T	0	0	0	0	0	0	0	0	0	0	1	Phosphoribosylaminoimidazole-succinocarboxamide synthase
1827478	C	T	0	0	0	0	0	0	0	0	0	0	1	N5-carboxyaminoimidazole ribonucleotide mutase
1839891	C	T	0	0	0	0	0	0	0	0	0	0	1	intergenic

1851908	G	A	0	1	0	1	0	0	0	0	0	0	0	intergenic
1859966	A	T	0	0	0	0	0	0	0	0	0	0	1	Ribonuclease 3
1860961	C	T	0	0	0	0	0	0	0	0	1	0	0	3-oxoacyl-ACP reductase
1864721	C	T	0	0	0	0	0	0	0	0	0	0	1	ATP-dependent DNA helicase RecG
1868378	G	T	0	0	0	0	0	0	0	1	0	0	0	DAK2 domain-containing protein
1868456	G	T	0	0	0	0	0	0	0	0	0	1	0	DAK2 domain-containing protein
1869093	C	T	0	0	0	0	0	1	0	0	0	0	0	hypothetical protein
1869410	G	A	0	0	0	0	0	0	0	0	0	0	1	DAK2 domain protein
1869584	A	T	1	0	0	0	0	0	0	0	0	0	0	DAK2 domain-containing protein
1870118	A	G	0	1	0	1	0	0	0	0	0	0	0	intergenic
1870140	A	G	0	0	0	0	0	0	0	0	1	0	0	intergenic
1888814	A	C	1	1	1	1	1	1	1	1	1	1	1	dihydroorotate dehydrogenase, electron transfer subunit
1902751	T	C	0	0	0	0	0	0	0	0	0	0	1	Multidrug resistance protein NorM
1907497	T	A	1	1	1	1	1	1	1	1	1	1	1	Carboxypeptidase
1925447	G	T	1	1	1	1	1	1	1	1	1	1	0	serine protease
1951931	T	G	0	0	0	0	0	0	1	0	0	0	0	aspartate aminotransferase
1962138	C	A	1	1	1	1	1	1	1	1	1	1	0	dihydrodipicolinate reductase
1964660	C	A	0	0	0	0	0	0	0	0	1	0	0	oxidoreductase, FAD-binding
1964679	G	C	0	0	0	0	0	0	0	0	1	0	0	oxidoreductase, FAD-binding
1964681	T	C	0	0	0	0	0	0	0	0	1	0	0	oxidoreductase, FAD-binding
1964690	T	C	0	0	0	0	0	0	0	0	1	1	0	oxidoreductase, FAD-binding
1967124	G	T	1	1	1	1	1	1	1	1	1	1	1	diguanylate cyclase
1967125	T	G	1	1	1	1	1	1	1	1	1	1	1	diguanylate cyclase
1967516	G	T	0	0	0	0	0	0	0	0	0	0	1	hypothetical protein
1968363	G	T	0	0	0	0	0	0	0	0	0	0	1	putative membrane protein YjcC
1977688	G	A	1	1	1	1	1	1	1	1	1	1	1	hypothetical protein
1981628	G	A	0	0	0	0	0	0	0	0	0	0	1	Histidinol-phosphate aminotransferase
1983548	C	T	0	0	0	0	0	0	0	0	0	0	1	3-dehydroquinate synthase
1993762	C	T	1	1	1	1	1	1	1	1	1	1	0	30S ribosomal protein S1
2000034	A	G	1	1	1	1	1	1	1	1	1	1	1	hypothetical protein
2002389	T	A	0	0	0	0	0	0	0	0	0	0	1	Bacterial Ig-like domain (group 3)
2005387	G	A	1	1	1	1	1	1	1	1	1	1	0	cell wall surface anchor protein
2027569	G	T	0	0	0	0	0	0	0	0	0	0	1	5-bromo-4-chloroindolyl phosphate hydrolysis protein
2050326	G	A	0	1	0	1	0	0	0	0	0	0	0	LacI family transcriptional regulator
2057115	G	A	1	1	1	1	1	1	1	1	1	1	0	PTS system mannose/fructose/sorbose family transporter subunit IIB
2066512	C	G	1	1	1	1	1	1	1	1	1	1	0	DNA-binding response regulator
2069051	G	A	1	1	1	1	1	1	1	1	1	1	0	hypothetical protein
2077570	C	T	0	0	0	0	0	0	0	0	0	0	1	undecaprenyl pyrophosphate phosphatase
2078186	T	C	1	1	1	1	1	1	1	1	1	1	1	intergenic
2078321	C	T	0	0	0	0	0	0	0	0	0	0	1	Diaminopimelate epimerase
2100758	A	G	1	1	1	1	1	1	1	1	1	1	1	hypothetical protein
2102049	G	C	1	1	1	1	1	1	1	1	1	1	1	cell division protein FtsZ
2102111	G	T	1	1	1	1	1	1	1	1	1	1	1	cell division protein FtsZ
2102356	A	G	1	1	1	1	1	1	1	1	1	1	1	cell division protein FtsA
2102392	A	G	1	1	1	1	1	1	1	1	1	1	1	cell division protein FtsA
2102408	A	G	1	1	1	1	1	1	1	1	1	1	1	cell division protein FtsA
2103381	G	C	1	1	1	1	1	1	1	1	1	1	1	cell division protein FtsA
2107969	G	T	1	1	1	1	1	1	1	1	1	1	0	phospho-N-acetylmuramoyl-pentapeptide-transferase
2110379	A	G	1	1	1	1	1	1	1	1	1	1	1	penicillin-binding protein
2112693	G	C	0	0	0	0	0	0	0	0	0	0	1	Ribosomal RNA small subunit methyltransferase H
2117415	C	A	1	1	1	1	1	1	1	1	1	1	1	hypothetical protein
2117497	C	A	1	1	1	1	1	1	1	1	1	1	1	intergenic
2117518	C	A	1	1	1	1	1	1	1	1	1	1	1	intergenic
2117519	G	C	1	1	1	1	1	1	1	1	1	1	1	intergenic
2117553	C	A	1	1	1	1	1	1	1	1	1	1	1	intergenic
2122990	C	A	1	1	1	1	1	1	1	1	1	1	1	daunorubicin resistance protein
2123017	C	T	1	1	1	1	1	1	1	1	1	1	1	daunorubicin resistance protein
2125910	T	C	0	0	0	0	0	0	0	0	0	0	1	hypothetical protein
2148046	T	G	0	0	0	0	1	0	0	0	0	0	0	hypothetical protein
2155950	T	C	1	1	1	1	1	1	1	1	1	1	0	TetR family transcriptional regulator

2156284	C	A	1	1	1	1	1	1	1	1	1	1	0	TetR family transcriptional regulator
2168566	C	T	1	1	1	1	1	1	1	1	1	1	0	GntR family transcriptional regulator
2170154	G	A	1	0	0	0	0	1	0	0	0	1	0	pyridoxal biosynthesis lyase PdxS
2171475	G	C	0	0	0	0	0	0	0	0	0	0	1	Phosphate acetyltransferase
2176134	C	A	0	0	0	0	0	0	0	0	0	0	1	intergenic
2176174	A	G	0	0	0	0	0	0	0	0	0	0	1	intergenic
2181537	G	C	0	0	0	0	0	0	0	0	0	0	1	putative heme peroxidase
2183621	A	C	1	1	1	1	1	1	1	1	1	1	1	ABC transporter permease
2185381	T	C	0	0	0	0	0	0	0	0	0	0	1	hypothetical protein
2193708	T	C	0	0	0	0	0	0	0	0	1	0	0	maltose phosphorylase
2200429	C	T	0	0	0	0	0	0	0	0	0	0	1	intergenic
2217358	C	A	0	0	0	0	0	0	0	0	0	0	1	intergenic
2221944	G	T	0	0	0	0	0	0	0	0	0	0	1	intergenic
2232920	A	G	0	0	0	0	0	0	0	0	0	0	1	putative oxidoreductase YdgJ
2239505	G	A	0	0	0	0	0	0	0	0	0	0	1	hypothetical protein
2254187	G	T	0	0	0	0	0	0	0	0	0	0	1	Internalin-J precursor
2274742	C	T	0	0	0	0	0	0	0	0	0	0	1	Regulatory protein Spx
2284586	A	C	0	0	0	0	0	0	0	0	0	0	1	intergenic
2288351	C	T	0	0	0	0	0	0	0	0	0	0	1	intergenic
2301377	T	C	0	0	0	0	0	0	0	0	0	0	1	hypothetical protein
2305320	C	T	0	0	0	0	0	0	0	0	0	0	1	hypothetical protein
2319161	C	T	1	1	1	1	1	1	1	1	1	1	0	LysR family transcriptional regulator
2328852	A	G	1	1	1	1	1	1	1	1	1	1	1	intergenic
2330456	C	T	1	1	1	1	1	1	1	1	1	1	0	ribosomal large subunit pseudouridine synthase D
2330803	T	C	1	1	1	1	1	1	1	1	1	1	1	Glyoxalase
2332568	A	C	1	1	1	1	1	1	1	1	1	1	1	intergenic
2338153	G	A	1	1	1	1	1	1	1	1	1	1	0	intergenic
2367071	G	A	1	1	1	1	1	1	1	1	1	1	0	Luciferase
2368498	C	A	1	1	1	1	1	1	1	1	1	1	0	amino acid ABC transporter ATP-binding protein
2374017	C	T	0	0	0	0	0	0	0	0	0	0	1	Sodium, potassium, lithium and rubidium/H(+) antiporter
2386011	T	C	1	1	1	1	1	1	1	1	1	1	0	amino acid antiporter
2417049	C	T	0	0	0	0	0	0	0	0	0	0	1	putative ion channel protein
2420004	G	A	1	1	1	1	1	1	1	1	1	1	1	NADH dehydrogenase
2459520	G	A	0	0	0	0	0	0	0	0	0	0	1	hypothetical protein
2461214	G	A	0	0	0	0	0	1	0	0	0	0	0	transcriptional regulator
2476700	T	C	1	1	1	1	1	1	1	1	1	1	0	ribonuclease R
2487091	C	T	0	0	0	0	0	0	0	0	1	0	0	intergenic
2489150	A	G	0	0	0	0	1	0	0	0	0	0	0	RNA polymerase sigma-54 factor
2489268	G	A	0	0	0	0	0	0	0	1	0	0	0	RNA polymerase factor sigma-54
2492076	C	T	0	0	0	0	0	0	0	0	0	0	1	Membrane protein YdfJ
2492861	A	G	1	1	1	1	1	1	1	1	1	1	1	membrane protein
2508044	C	T	0	0	0	0	0	0	0	0	0	0	1	UDP-glucose 4-epimerase
2520895	T	C	1	1	1	1	1	1	1	1	1	1	1	excinuclease ABC subunit B
2533867	T	C	1	1	1	1	1	1	1	0	0	1	0	cardiolipin synthetase
2534353	G	T	0	0	0	0	0	0	0	1	0	0	0	cardiolipin synthetase
2574363	G	C	0	0	0	0	0	0	0	0	0	0	1	Peptide chain release factor 1
2590379	C	T	0	0	0	0	0	0	0	0	0	0	1	Internalin B precursor
2590403	C	T	0	0	0	0	0	0	0	0	0	0	1	Internalin B precursor
2590910	A	G	0	0	0	0	0	0	0	0	0	0	1	Internalin B precursor
2601174	G	A	0	0	0	0	0	0	0	0	0	0	1	Dipeptide-binding protein DppE precursor
2609736	A	T	1	1	1	1	1	1	1	1	1	1	0	ABC transporter ATP-binding protein
2609810	C	A	1	0	0	0	0	0	0	0	0	0	0	ABC transporter ATP-binding protein
2611568	C	T	1	0	0	0	0	0	0	0	0	0	0	sensor histidine kinase
2613386	C	A	0	0	1	0	0	0	0	0	0	0	0	formate dehydrogenase accessory protein
2615226	C	A	0	0	0	0	0	0	0	0	0	0	1	Putative formate dehydrogenase
2630235	C	T	0	0	0	0	0	0	0	0	0	0	1	Glyoxal reductase
2633776	C	T	1	1	1	1	1	1	1	1	1	1	0	intergenic
2653919	C	T	0	0	0	0	0	0	0	0	0	0	1	50S ribosomal protein L3
2660594	G	A	1	0	0	0	0	0	0	0	0	0	0	pyridine nucleotide-disulfide oxidoreductase
2662797	G	A	0	0	0	0	0	0	0	0	0	0	1	Heptaprenyl diphosphate synthase component 2
2676448	A	G	1	1	1	1	1	1	1	1	1	1	0	hypothetical protein
2676609	T	C	1	1	1	1	1	1	1	1	1	1	0	hypothetical protein

2679168	G	A	0	0	0	0	0	0	0	0	1	0	0	elongation factor G
2686938	G	T	0	0	1	0	0	0	0	0	0	0	0	Transketolase
2687916	C	T	0	0	0	0	0	0	0	0	0	0	1	Transketolase
2693341	C	T	1	1	1	1	1	1	1	1	1	1	0	intergenic
2694633	G	A	0	0	0	0	0	0	0	0	0	0	1	Transcriptional regulator MtlR
2700308	C	G	1	1	1	1	1	1	1	1	1	1	1	intergenic
2716903	T	C	0	0	0	0	0	0	0	0	1	0	0	magnesium-translocating P-type ATPase
2727710	G	A	1	1	1	1	1	1	1	1	1	1	0	intergenic
2731975	G	A	0	0	0	0	0	0	0	0	0	0	1	DNA polymerase III subunit tau
2735425	G	A	0	0	0	0	0	0	0	0	0	0	1	hypothetical protein
2736250	A	G	1	0	0	0	0	1	0	0	0	1	0	intergenic
2740273	G	A	0	0	0	0	0	0	0	0	0	0	1	hypothetical protein
2743464	G	A	0	0	0	0	0	0	0	0	0	0	1	ATP-binding/permease protein CydD
2747309	G	T	0	0	0	0	0	0	0	0	0	0	1	intergenic
2757086	G	A	1	1	1	1	1	1	1	1	1	1	0	hypothetical protein
2780022	G	T	0	1	0	1	0	0	0	0	0	0	0	seryl-tRNA synthetase
2794455	G	A	1	1	1	1	1	1	1	1	1	1	1	DNA topoisomerase III
2796160	C	T	0	0	0	0	0	0	0	0	0	0	1	DNA topoisomerase 3
2802980	A	T	0	0	0	0	0	0	1	0	0	0	0	aryl-phospho-beta-D-glucosidase
2807726	G	T	0	0	1	0	0	0	0	0	0	0	0	RpiR family phosphosugar-binding transcriptionalregulator
2831990	C	T	0	0	0	0	0	0	0	0	0	0	1	Catalase
2832229	T	G	1	1	1	1	1	1	1	1	1	1	1	Catalase
2845800	C	A	0	0	0	0	0	0	0	0	0	0	1	hypothetical protein
2849196	G	T	0	0	0	0	0	0	0	0	0	1	0	hypothetical protein
2849648	C	A	1	1	1	1	1	1	1	1	1	1	0	intergenic
2859063	A	T	0	0	0	0	0	0	0	0	0	0	1	3-oxoacyl-[acyl-carrier-protein] reductase FabG
2868418	A	G	1	1	1	1	1	1	1	1	1	1	1	cell wall surface anchor family protein
2876321	A	G	0	0	0	0	0	0	0	0	0	0	1	intergenic
			**168**	**155**	**157**	**155**	**155**	**158**	**157**	**161**	**171**	**164**	**230**	**Total 377, 83 common, 57 intergenic**

Supplementary Table 4. Position and respective designation of gene harboring single nucleotide polymorphisms. The SNP was calculated based on the reference nucleotide in genomically closest strain *L. monocytogenes* F2365. (Key: 1: SNP present; 0: No SNP). Comparatively higher SNP frequencies observed upstream/downstream region of transposases are marked in red.
